# AppleQSM: Geometry-Based 3D Characterization of Apple Tree Architecture in Orchards

**DOI:** 10.34133/plantphenomics.0179

**Published:** 2024-05-01

**Authors:** Tian Qiu, Tao Wang, Tao Han, Kaspar Kuehn, Lailiang Cheng, Cheng Meng, Xiangtao Xu, Kenong Xu, Jiang Yu

**Affiliations:** ^1^ School of Electrical and Computer Engineering, Cornell University, Ithaca, NY, USA.; ^2^ Institute of Statistics and Big Data, Renming University of China, Beijing, China.; ^3^ Department of Ecology and Evolutionary Biology, Cornell University, Ithaca, NY, USA.; ^4^ School of Integrative Plant Science, Cornell University, Ithaca, NY, USA.; ^5^ Center for Applied Statistics, Renmin University of China, Beijing, China.; ^6^ School of Integrative Plant Science, Cornell University, Ithaca, NY, USA.

## Abstract

The architecture of apple trees plays a pivotal role in shaping their growth and fruit-bearing potential, forming the foundation for precision apple management. Traditionally, 2D imaging technologies were employed to delineate the architectural traits of apple trees, but their accuracy was hampered by occlusion and perspective ambiguities. This study aimed to surmount these constraints by devising a 3D geometry-based processing pipeline for apple tree structure segmentation and architectural trait characterization, utilizing point clouds collected by a terrestrial laser scanner (TLS). The pipeline consisted of four modules: (a) data preprocessing module, (b) tree instance segmentation module, (c) tree structure segmentation module, and (d) architectural trait extraction module. The developed pipeline was used to analyze 84 trees of two representative apple cultivars, characterizing architectural traits such as tree height, trunk diameter, branch count, branch diameter, and branch angle. Experimental results indicated that the established pipeline attained an *R*^2^ of 0.92 and 0.83, and a mean absolute error (MAE) of 6.1 cm and 4.71 mm for tree height and trunk diameter at the tree level, respectively. Additionally, at the branch level, it achieved an *R*^2^ of 0.77 and 0.69, and a MAE of 6.86 mm and 7.48° for branch diameter and angle, respectively. The accurate measurement of these architectural traits can enable precision management in high-density apple orchards and bolster phenotyping endeavors in breeding programs. Moreover, bottlenecks of 3D tree characterization in general were comprehensively analyzed to reveal future development.

## Introduction

Apples, temperate fruit renowned for their delectable flavor and health-boosting properties, are packed with vitamins, minerals, and dietary fiber that shield the human body from oxidative stress and chronic illnesses [1]. Apple production is economically vital globally and in the United States, where it has a farm value of $3.2 billion in 2022 [2]. The sustainable growth of the apple industry hinges on the development and management of apple trees with optimal architectural traits, as these traits substantially influence the trees’ vegetative growth, fruiting potential, and environmental interactions. For instance, a tree’s height affects the light exposure to its lower sections, impacting the yield and quality of the fruit in commercial orchards, while the trunk diameter determines optimal crop load. Therefore, it is imperative for growers to accurately assess tree architectural traits to proficiently manage orchards for maximal productivity and superior quality.

Apple tree characterization has been dependent on manual measurements and observations, which include visual inspection and the use of tape measures or calipers for assessing traits. These methods are laborious and subjective and often fail to capture the intricate and diverse nature of tree architecture, which is critical for fruit production. For instance, visual inspection may not discern minor variations in branch angles, lengths, or diameters that influence the distribution of fruiting sites and the tree’s overall fruiting capacity. Furthermore, complex branch and tree morphology may present challenges in placing conventional tools (e.g., angle rulers) for measurements in the field. Optical sensing technologies (especially imaging) are gaining traction due to their noninvasive, versatile, and cost-effective attributes. These technologies furnish detailed insights into plant architecture as well as physiological and biochemical properties [3–5]. Consequently, there is an escalating interest in the development of cutting-edge imaging technologies and machine learning (ML) methods for more precise and efficient plant trait characterization. Leveraging these technologies could surmount the constraints of conventional methods, yielding a holistic comprehension of tree architectural traits, which is essential for the enhanced management and optimization of fields and orchards.

2D imaging systems have been broadly used to characterize trees and tree crops [6]. Over time, there has been a noticeable transition from reliance on visual and geometric methods [7–14] toward the adoption of ML-based approaches [12,13,15–19]. This shift is due primarily to the substantial improvements of model accuracy and robustness that deep learning (DL) models have offered in recent years [3,20]. However, occlusion and perspective ambiguity issues dramatically limit the use of 2D imaging methods, particularly in the field, presenting major challenges in tree and tree crop characterization. To overcome these challenges, researchers have extensively studied and improved three-dimensional (3D) sensing technologies, such as multi-view 3D reconstruction systems and LiDAR sensors such as terrestrial laser scanner (TLS), leading to the active development of 3D data processing for tree trait characterization [21,22]. These pipelines typically involve four main steps: data acquisition, preprocessing, segmentation, and trait extraction [23]. Among these pipelines, point cloud is a commonly used 3D data representation because of its effective representativeness of object geometry and topology, efficient data storage, and scalable processing [24].

From a data processing standpoint, point cloud-based pipelines for tree modeling are broadly categorized into geometry- and learning-based approaches. A prominent tree modeling method within the geometry-based paradigm is quantitative structural modeling, which aims to reconstruct a quantitative structure model (QSM) of trees by capturing essential topology, geometry, and volume properties, including branch quantity, length, volume, angle, and size distribution. The development of QSM methods has been an active research area for the forestry community, and these methods have demonstrated impressive performance in forest volume estimation [25,26], above-ground biomass (AGB) measurement of different tree species [27], and tree species identification [28,29] and forest radiation transmission simulation [30]. Existing QSM methodologies can be classified into two primary categories: segmentation-based and skeleton-based. Segmentation-based approaches entail initial segmentation of the tree point cloud into smaller subsets, followed by procedural connection to reconstruct the tree’s topological structure [31–34]. An illustrative example is TreeQSM [34], which hierarchically transforms point clouds into a collection of cylinders to characterize tree topology, geometry, and volume. However, a notable limitation of segmentation-based methods is their susceptibility to input data quality, potentially compromising robustness when faced with issues such as outliers or missing data due to occlusions. In contrast, skeleton-based methods directly extract skeletal curves from raw input point clouds [35–38]. AdQSM [39], a novel QSM model based on AdTree [40], employs a skeleton-based approach and a distance-weighted cylinder fitting to precisely reconstruct tree branches from individual point clouds.

Learning-based processing pipelines utilize extensive 3D point cloud data and employ ML or DL models to extract robust features and relationships, yielding more dependable, generalizable, and precise outcomes. There is a growing body of research focused on developing 3D ML/DL models for organ-level segmentation, which have proven to surpass traditional methods in segmentation accuracy, thereby enhancing characterization performance across diverse crops, including tree [7,41–44] and field crops [45–47]. Supervised training of a reliable ML/DL model demands a vast amount of annotated data, posing substantial challenges for 3D point cloud analyses. To mitigate the challenge of extensive data annotation, weakly supervised methods (e.g., Eff-3DPSeg) were proposed to reduce the data annotation burden for accurate and robust model training [48]. Although notable performance was observed, challenges remained in the selection of representative points for annotation and the quality (e.g., point cloud completeness and noises) of raw point clouds for processing. Further research is required to address these limitations for the adoption of nonsupervised methods for efficiently training reliable models for 3D point cloud analyses.

For apple tree characterization, previous research largely focused on particular facets, such as tree branch detection [49], fruit detection [42,50], and leaf area analysis [51], rather than a holistic examination of traits from the branch to tree levels. A recent study first explored the use of TreeQSM for extracting branch information of apple trees, examining the efficacy of translating analysis methods developed for forestry to agriculture where assumptions in tree architecture and density would be different [49]. Experimental results showed the best accuracy of 88% in detecting and counting the first-order (i.e., primary) branches and that of 92.57% in estimating the number of primary branches, demonstrating certain successes of using TreeQSM and TLS data for the characterization of fruit trees such as apple trees. However, the study highlighted several major limitations of using TreeQSM for apple tree characterization. First, high-resolution TLS devices and calm weather were needed to resolve granular details of apple trees that can be characterized by QSM. Second, TreeQSM used random seeds to initiate the tree reconstruction, presenting large variation and error in tree trait measurements. This issue dramatically hinders the use of TreeQSM (or other QSMs involving reconstruction randomness) for tree crop characterization in agricultural research and management where consistent measurements are required to understand tree growth and guide field operations. It should be noted that all these results and findings were obtained from apple trees without trellising, which shared considerable similarities with natural trees and simplified additional challenges because of data quality issues (e.g., incomplete point clouds due to high occlusions) caused by the high tree density in trellising systems. Therefore, new methodologies must be developed to address the challenges of characterizing apple trees for management applications in modern orchards with trellised tree training.

The study’s primary contribution was to develop a comprehensive characterization pipeline (referred to as AppleQSM hereafter), leveraging TLS point cloud data, for the quantitative analysis of architectural traits for tree fruit crops that are trained using modern practices such as high-density apple orchards. While acknowledging the existence of some parallels between this work and established QSM methods, a detailed comparative analysis was conducted to scrutinize bias and the extent of randomness, illuminating the distinct advantages that our methodology offers when applied within the realm of agricultural research and applications. Additionally, this study aimed at establishing essential performance benchmarks and raising inherent challenges associated with data quality. Notably, the study stands out as one of the first endeavors in tree crop research to critically examine a fundamental bottleneck within the domain of 3D tree QSM, specifically from the perspective of data quality. By doing so, it not only identifies the core issues but also offers potential pathways to address these data-related challenges. This contribution marks a significant step forward in advancing the field of tree crop research, promising valuable insights and directions for future investigations in agricultural science and technology. The datasets and pipeline codebase in this study are publicly available to facilitate further research and promote technology adoption in production management. The specific objectives included (a) the development of a graph-based characterization pipeline encompassing tree structure segmentation and tree- and branch-level analysis, (b) an evaluation of the pipeline’s performance at both tree and branch levels, and (c) an investigation into the principal constraints affecting characterization performance to shed light on potential avenues for future advancements.

## Methods

**Fig. 1.**
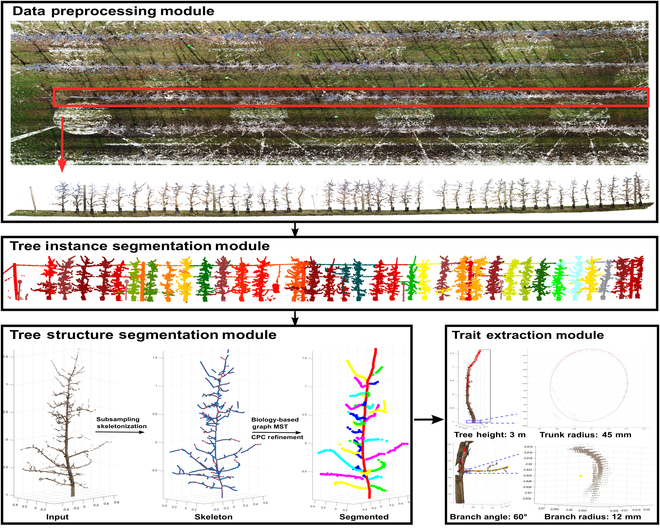
AppleQSM workflow. The ROI was manually selected from the raw point cloud data and denoised using the SOR in CloudCompare. The tree instance segmentation module takes the ROI as input and segments each tree as an individual point cloud. Each individual tree was processed by the tree structure segmentation module to separate the trunk and branches for trait extraction.

AppleQSM was developed to characterize apple tree architecture using full-view point clouds. It consisted of four modules: data preprocessing module, tree instance segmentation module, tree structure segmentation module, and architectural trait extraction module (Fig. 1). The meaning of symbols and the parameter values getting involved in the pipeline were summarized in Tables S2 and S3.

### Data preprocessing module

Preprocessing of these registered point clouds involved the removal of extraneous data. Given the extensive field of view of the laser scanner (360° by 300° per location) and the multiple scanning locations, the collected data often included irrelevant objects such as neighboring row trees, trellis wires, and tree covers. To focus on the row of interest, the region of interest (ROI) was manually selected to contain only the point clouds associated with trees in the desired row. Raw point data were denoised using the statistical outlier removal (SOR) with the default parameters implemented by CloudCompare (CloudCompare, version 2.11.3). In addition, point data related to tree covers and trellis wires were manually removed from the ROI.

### Tree instance segmentation module

For each tree row, individual apple trees were automatically segmented using a computationally efficient algorithm based on geometric features [52]. The algorithm iteratively searched for linear units, defined as connected point clusters over a cluster-level linearity threshold, from input point clouds. Linear units generally represent stems or branch segments. The algorithm then assembled linear units into individual trees based on connectivity and topology. This approach was capable of more robustly identifying small stems and thin branches compared with existing tree segmentation methods [53].

### Tree structure segmentation and refinement module

#### Tree skeletonization

Structure aware downsampling: The point cloud of an individual apple tree typically contained 700,000 points, requiring data downsampling to ensure tractable computation in successive analyses. Numerous sampling techniques have been devised to address the challenges associated with handling vast amounts of data [54,55]. However, these methods often exhibit a deficiency in incorporating structural awareness during the downsampling process. A Hilbert curve (HC)-based downsampling method was used to downsample tree point clouds with well-preserved topological structure and geometric features due to the locality-preserve property of HC (Fig. S2) [56,57]. In HC *H* was a limit of a sequence of the Hilbert space-filling curves. The *k*th order Hilbert space-filling curve *H_k_* was a bijection between a partition of [0, 1] and [0, 1]*^d^* in *d*-dimensional space. The tree point cloud $P={\left\{p_{i}\right\}}_{i=1}^{n}$ was normalized into [0, 1]^3^, and the bijection between ${\left\{c_{j}^{\prime }\right\}}_{j=1}^{2^{3k}}$ and ${\left\{c_{j}\right\}}_{j=1}^{2^{3k}}$ was calculated where $c_{j}^{\prime }$ and *c_j_* were associated blocks in [0, 1] and [0, 1]^3^, respectively. A point *p_i_* specific to block *c_j_* was mapped to the center of the block $c_{j}^{\prime }=H_{k}^{-1}\left(c_{j}\right)$. A histogram was drawn for the mapped points, and *N_h_* data points were selected from each nonempty bin (Eq. 1).

$$N_{h}=\frac{N_{S}}{\#\text{Bins}}$$ (1)

*N_S_* is the target number of points after downsampling, and the number of bins was determined by the order of the HC. Intuitively, the selected data points were roughly equally distributed in the original space and thus capable of preserving the tree structure effectively. The downsampled point cloud *P_D_* was used for the next step.

Laplacian-based skeletonization: A Laplacian-based contraction method was used to extract the tree skeleton from the denoised and downsampled point cloud *P_D_* because of its robustness to noise and missing points [58]. The skeletonization was achieved by geometric contraction and topological thinning. In the geometric contraction, the original point cloud was contracted by iteratively optimizing a linear system. In the topological thinning, the contracted point cloud was sampled using farthest point sampling (FPS) with a resolution of ϵ to produce a sampled point cloud. The selection of the sphere radius could critically impact the resolution of the final skeleton points and therefore the accuracy of architectural trait extraction. In this study, for enhanced generalization amidst tree variability, the parameter ϵ was adaptively established based on the length of the bounding box’s diagonal of the original tree point clouds. The connection of the sampled point cloud was built by inheriting the sample neighbors. The final skeleton *P*^′^ was obtained by collapsing unnecessary edges until no triangles existed.

Skeleton connectivity refinement: Although the established connections within *P*^′^ directly indicated a topological relationship, numerous inaccuracies existed, including (a) discontinuity between proximate points, (b) false continuity between distant points, and (c) circular linkages among points. These inaccurate connections would present significant challenges in tree structure segmentation such as tree trunk identification. To address these issues, a biology-aware (BA) refinement method was developed to improve the skeleton connectivity by using biological cues (Fig. S3). An intuitive observation was that the thickness gradually diminished along the tree trunk or branch. Based on this, the thickness was used to provide a local constraint for correcting skeleton point connectivity.

The BA refinement method first corrected the discontinuity of proximate points and false continuity between distant points using a distance constraint: a pair of points were connected only if their Euclidean distance was smaller than a distance threshold *d_th_*. The corrected skeleton points were subsequently converted into a weighted graph *G_W_*, in which vertices were the skeleton points and edges represented the point connectivity. The developed refinement method considered both the thickness and Euclidean distance information in the edge weight calculation (Eqs. 2 and 3). The minimum spanning tree (MST) algorithm was applied on *G_W_* to remove redundant and cyclic connections, and the MST with the greatest number of vertices was used as the coarse tree skeleton *P_S_*. While the thickness guarantees local smoothness (thickness decreasing pattern) and continuity (connections among proximate points), the Euclidean distance term constrains the connections globally by guiding the MST algorithm to find the topologically simplest graph. It should be noted that the thickness of a point would be ideally calculated as the diameter of a plant tissue. However, due to practical computation challenges, the thickness was approximated by point density in this study. This was because thicker regions would reflect more laser pulses and yield a higher point density that can be approximated as the reciprocal of the Euclidean distance to its *K* nearest neighbors in *P* (Eq. 3).

$$e_{i,j}=\alpha \left(\frac{1}{m_{i}}+\frac{1}{m_{j}}\right)+\left(1-\alpha \right)d_{i,j}$$ (2)

$$m_{i}=\frac{1}{\frac{1}{k}\sum_{j=1}^{k}\;d_{i,j}}$$ (3)

where *e*_*i*,*j*_ represents the weight of the edges between the *i*th and *j*th points, *m_i_* is the estimated density of the *i*th point, and *d*_*i*,*j*_ is the 3D Euclidean distance to its *j*th nearest neighbor. *α* is the weight to balance the local smoothness due to thickness and the length information. The first and second terms in *e*_*i*,*j*_ are already normalized with respect to itself to avoid any scaling effects. The rationale for using the reciprocal of the thickness is to align with the MST algorithm optimization goal of minimizing the connection cost. The following trunk and branch segmentation and refinement were achieved on *P_S_* rather than the input point cloud.

#### Trunk and branch segmentation

The trunk of a tree refers to the main wooden axis of a tree in botany, which is usually the longest axis of an apple tree. According to the botanical definition, the coarse trunk skeleton was identified by finding the longest path (i.e., the maximal weight) in *P_S_*. This required the recalculation of weights in *P_S_* using Eq. 4 to generate $P_{S}^{\prime }$. For each point in $P_{S}^{\prime }$, its path to the trunk skeleton root point (i.e., the lowest point in $P_{S}^{\prime }$) and the associated path weight were found using the Dijkstra’s algorithm (Eq. 5).

$$e_{i,j}^{\prime }=\alpha \left(m_{i}+m_{j}\right)+\left(1-\alpha \right)d_{i,j}$$ (4)

$$W_{k}=\sum_{e^{\prime }\in E_{k}}\;e_{i,j}^{\prime }$$ (5)

where $e_{i,j}^{\prime }$ represents the updated weight of the edges between the *i*th and *j*th points, and *W_k_* is the weight of the path from the *k*th point to the root point in $P_{S}^{\prime }$.

Trees inherently possess a recursive structure, meaning their structural components can be similarly defined across varying levels. For instance, the tree trunk, being the longest wooden structure that originates from the junction of the tree and ground, is mirrored by a primary branch, which forms the longest wooden structure among all the tissues that grow from the trunk–branch junction. Based on this, the developed trunk identification method could be iteratively used to find branches at all levels (e.g., primary and secondary). The key to this iterative process was to correctly cluster points of all tissues belonging to individual branches at the next level after identifying the trunk/branch at the current level.

The identification of the primary branch was used as an example to describe this iterative process. After tree trunk identification, the trunk skeleton points were used to form a tube space where cross sections were set by using the trunk skeleton points as centers with a constant radius *R* from the lowest to the highest points [59]. The tube space was used to retain branch points close to the tree trunk, resulting in a simplified trunk–branch structure for detecting primary branch origin points. The trunk skeleton points were removed from the retained points, and the resultant points were clustered as the origin point groups of individual primary branches using 3D density-based spatial clustering of applications with noise (DBSCAN). In some cases, skeleton points of nonprimary branches close to the trunk might be included in the tube space because of complex branch patterns, leading to incorrect primary branch origin clusters. Such incorrect primary branch origin clusters were identified by a projection distance constraint: Points in the cluster were used to fit a 3D line with extended projection to find a possible intersection between that cluster and the trunk. The length of the extended line was compared to a predefined threshold *d_ext_* to keep valid primary branch origin clusters for successive processes.

For each valid primary branch origin cluster, the BA graph algorithm was used to identify the optimal MST (i.e., the MST with the greatest number of vertices) as the entire branch. Then, the primary branch was detected by finding the longest path in this MST (Eqs. 3 and 4).

While most primary branch points were assigned to a unique primary branch, several skeleton points could be selected for multiple primary branches. The multiple selection phenomenon mostly occurred if two branch origin clusters were close to each other. A maximum direction matching (MDM) method was developed to address this issue (Fig. S4). The method was inspired by the biological principle that branches typically have a smooth growing direction between their segments. The spectral clustering algorithm was used to group points that were (a) selected multiple times and (b) close to each other into *k*^′^ spectral clusters, where *k*^′^ was the number of selections for each point. Denote branch origin cluster points as *PB* = {*PB*_1_, *PB*_2_, …, *PB_k_*} and spectral cluster points as *PS* = {*PS*_1_, *PS*_2_, …, *PS_m_*}. For each branch origin cluster and spectral cluster pair, *PB_t_* and *PS_k_* with the minimum Euclidean distance between these two clusters were identified. Specifically, two 3D vectors were sequentially generated among *PB*_*t*−1_, *PB_t_*, and *PS_k_*, and the angle between these two vectors was computed to represent the growing direction. The spectral clusters were uniquely matched to branch origin clusters with the smoothest growing direction (i.e., the minimum angle).

Due to the incomplete original point cloud data, the segmented trunk and branch skeleton points are not guaranteed to present topological correctness and centeredness. Therefore, a skeleton refinement operation was used to obtain improved skeleton points.

#### Trunk and branch refinement

The cylindrical prior constraint (CPC)-based method was used to optimize the skeleton points of identified tree trunk and primary branches [60]. The CPC-based optimization employed two key constraints by exploiting the cylindrical shape prior to achieving topologically correct and well-centered skeleton points: (a) the *L*_1_ local median constraint to ensure the centeredness of the median points in a local point cluster and (b) the equidistant constraint to reinforce the median point close to the cylindrical axis of symmetry (Eq. 6).

$$v=\text{argmin}\sum_{i=1}^{m}\;|v-x_{i}|+{\lambda \sigma }^{2}$$ (6)

where *v* is the optimized local median, $X={\left\{x_{i}\right\}}_{i=1}^{n}$ is a local point set, *σ* is the variance of point-wise distance between *X* and the current *v*, and *λ* is a hyperparameter that balances these two constraint conditions.

For each skeleton point in *P_S_*, a local region was selected from the input points *P* by using the skeleton point as the center with a constant radius *R*^′^. This local region was further divided into cross sections sequentially (see points in different colors in Fig. S5). The CPC-based optimization was applied to each cross section to refine the skeleton point as the center of the cross section. Additionally, the radius of the cross section could be computed as the average Euclidean distance from the center to all points in the cross section. A local center correction operation (i.e., RANSAC algorithm) was applied within each local region to filter out the incorrect skeleton points that deviated from the central axis. To further enhance the smoothness of the skeleton points, a semi-global center correction operation (i.e., RANSAC algorithm) was applied to every *K*_1_ consecutive skeleton points. It was found that this semi-global center correction operation is considerably helpful for sparse areas. Finally, a cubic smoothing spline was fit using inlier centers and *M* points were evenly sampled from the spline as the refined skeleton points. The associated radius of sampled points was determined from the average radius of *N* nearest points.

### Architectural trait extraction module

Architectural traits important to tree crop load management were extracted using the input points *P* and refined tree trunk and branch skeletons at both the tree and branch levels. The tree-level traits included tree height and trunk diameter, and the branch-level traits included branch diameter and angle with respect to the tree trunk (i.e., branch inclination angle).

Tree-level architectural traits: Tree height was obtained by subtracting the *Z* value of the lowest point from the highest point in the refined trunk and adding 60 cm to account for the removed black cover. The trunk diameter was estimated using the bottom cross section consisting of points that were selected from the input point cloud *P* with the *z*-axis coordinate that is below a specific height. This height was determined by a constant value *C_z_* to the *z*-axis coordinate of the lowest point. To separate the trunk points from potential bottom branch points, a 3D DBSCAN clustering algorithm was applied to the bottom cross section. The largest group of points was considered the trunk points and was used for robust 2D ellipse fitting. The trunk radius was calculated as the average length of the major and minor axis of the fitted ellipse.

Branch-level architectural traits: Branch diameter and branch inclination angle were determined in an average fashion in order to minimize measurement errors. For each branch, the branch skeleton points were sorted based on their Euclidean distance to the trunk, and a local trunk segment was generated by selecting the refined trunk skeleton points that lie within the branch. A 3D trunk vector was generated by the RANSAC 3D line fitting algorithm, and branch vectors were produced between each neighboring branch skeleton points for branch inclination angle calculation. The final branch inclination angle was measured as the average 3D angle between the first *K*_2_ branch vectors and the sliced trunk (Eq. 7). Branch diameter was computed as the average of the radius of the first branch *K*_3_ skeleton points.

$$\theta =\frac{1}{K_{2}}\sum_{i=1}^{K_{2}}\text{arc}\;\cos\frac{V_{bi}\cdot V_{t}}{∥\;V_{bi}\;∥\;∥\;V_{t}\;∥}\;$$ (7)

where ‖ ⋅ ‖ is the norm of a vector, *V_bi_* is *i*th branch vector, and *V_t_* is the local trunk vector. The rationale for using the local trunk vector for branch inclination angle calculation is the observation that the tree trunk is typically not straight-up, meaning that a global trunk vector might not well represent the local trunk region and lead to inaccuracy in the computed inclination angle.

## Experiments

### Experiment orchard and field data collection

**Fig. 2.**
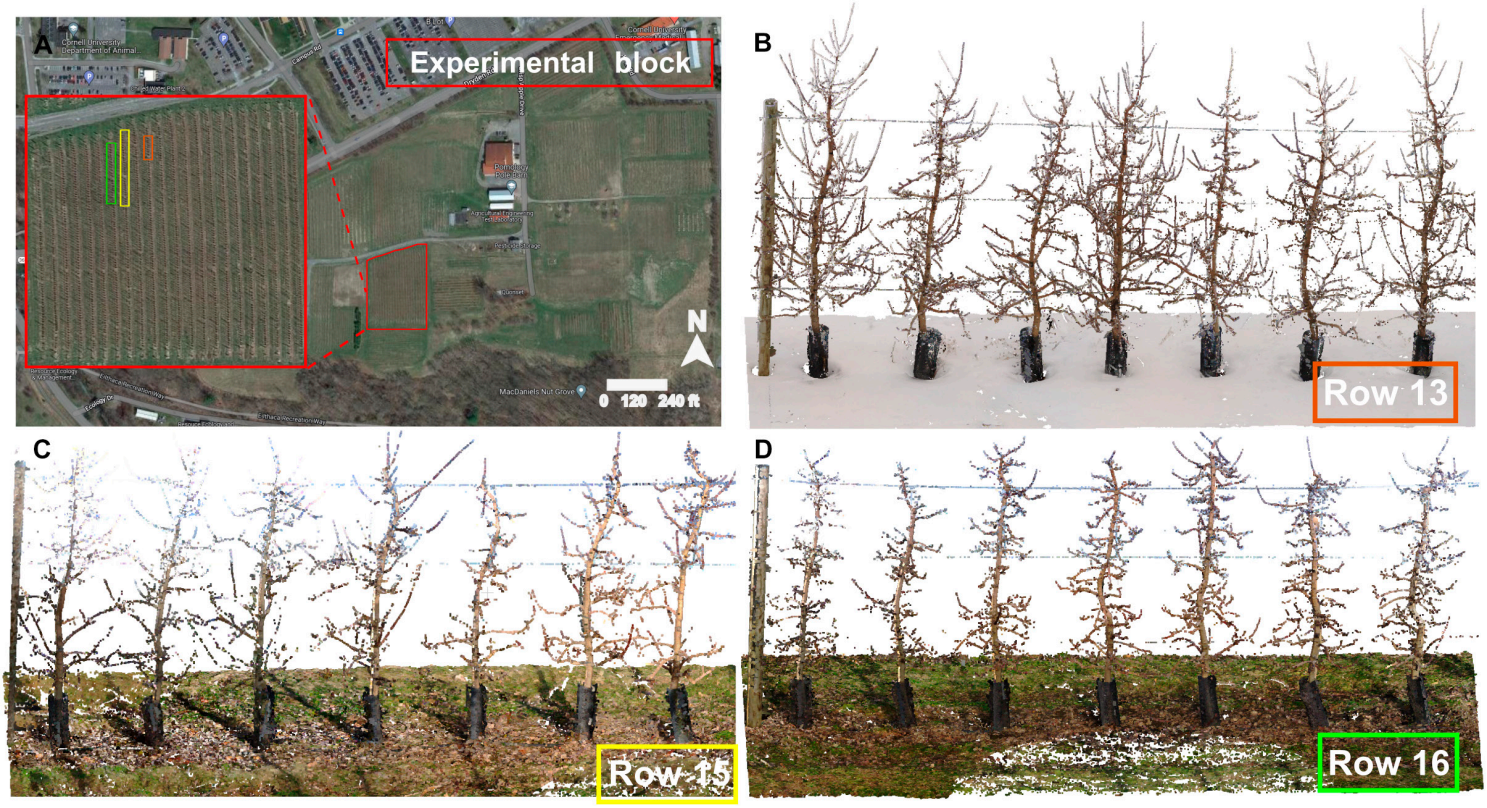
Experimental field and collected point cloud representatives. Experiments were conducted in nine blocks in three rows in Cornell Orchard.

Mature apple trees grown at Cornell Orchards (latitude: 42.445 N, longitude: 76.462 W) in Ithaca, NY, USA, were used in this study (Fig. 2). These trees were planted at a spacing of 3.66 m (12 feet) by 0.91 m (36 inches) in 2011 and trained in the tall spindle system. A total of 84 apple trees from three tree rows were used for data acquisition and characterization, including row 13 (9 trees) and 15 (41 trees) of “NY1” on M9 rootstock and row 16 (34 trees) of “NY2” on B.9 rootstock (Table 1).

**Table 1.** Summary of apple trees and point cloud data from the three crop rows used in this study

Row ID	Cultivar	#Trees	Age	Height (cm)			Trunk diameter (mm)			#Point cloud per tree		
				Min	Max	Mean	Min	Max	Mean	Min	Max	Mean
13	NY1 (SnapDragon)	9	12	322.58	386.08	355.04	46.00	60.50	55.61	662,830	1,363,933	912,007
15	NY1 (SnapDragon)	41	12	241.30	340.36	313.10	42.00	68.00	53.40	130,951	2,129,178	481,187
16	NY2 (RubyFrost)	34	12	276.86	355.6	312.27	42.5	68.00	56.78	280,998	680,007	475,853

As this study focused on tree architecture characterization, data acquisition trials were conducted during the offseason (three data collections from February to April 2022) with the maximal visibility of tree trunks and branches. A TLS (FARO Focus S350, FARO Technologies Inc., Lake Mary, FL, USA) with a 360° (horizontal) by 300° (vertical) scanning view was used to collect colorized point clouds of the apple trees (Fig. 2B). The scanner was mounted on a tripod at approximately 1.5 m above the ground and configured with a resolution of 6.1 mm at 10 m. The scanning of row 13 was meant to provide optimal data quality by maximizing the overlapping between neighboring scans, which was a trial to understand the trade-off between the number of scans and the data quality. In the following data collection for rows 15 and 16, the scanning positions were designed to maximize the scanning efficiency and data quality simultaneously, where the distance of two neighboring scans is around 10 m. In addition, scan references were strategically deployed in the field to improve the point cloud quality (Fig. S6).

### Pipeline evaluation

#### Quantitative metric

To evaluate the performance of the developed pipeline, reference measurements were obtained for all the traits using the protocols by the apple research community and industry (Table 2). Tree height and trunk diameters were measured for all 84 apple trees in this study, whereas branch diameter and inclination angle were measured for 106 branches from 9 apple trees in row 13. In addition, the number of branches was manually counted from collected point clouds using CloudCompare (CloudCompare, version 2.11.3).

**Table 2.** The ground-truth field measurements and the corresponding measurement protocol. A visual illustration of the branch diameter and inclination angle measurement in Fig. S7.

Architectural trait	Measurement protocol	#Samples
Tree height	The vertical distance from the highest point of the tree to the ground	84 trees
Trunk diameter	The average of two orthogonal measurements with a caliper at 5–10 cm above the black cover	84 trees
Branch diameter	A single measurement with an equilifruit disc (Valent LLC, USA) at 5 cm away from the trunk–branch junction	106 branches from 9 trees in row 13
Branch inclination angle	A single measurement with a digital angle finder (CamRad 82305, Guilin GemRed Sensor Technology Co., Ltd., China) in the crotch as close to the trunk and branch as possible	106 branches from 9 trees in row 13

Robust linear regression analyses were performed between pipeline-extracted measurements and reference values. Root mean square error (RMSE) and the coefficient of determination (*R*^2^) were used as metrics. In addition, mean absolute error (MAE) and mean absolute percentage error (MAPE) were calculated to comprehensively evaluate the accuracy of the developed pipeline. All analyses and calculations were conducted using MATLAB (version R2022a). While the pipeline was developed using a laptop with Intel Core i7-10870H with CPU running at 2.20 GHz, it is compatible with any laptops that support the specified MATLAB.

#### Comparison with TreeQSM and AdQSM

The latest implementation of TreeQSM (version 2.4.1) and AdQSM (version 1.7.5) was applied to characterize the apple trees under investigation, with a specific focus on quantifying the number of primary branches. To determine the optimal input parameters for TreeQSM, a meticulous exploration of critical parameters was conducted, including *PatchDiam1*, *PatchDiam2Min*, and *PatchDiam2Max*, among others. The search process encompassed 12 distinct parameter combinations generated by the *define*_*input* function, as recommended by established guidelines [61], for each tree. Within this search, five independent models were generated for each unique parameter combination. The combination that yielded the most favorable QSMs, as determined by the default average cylinder point-model distance, was ultimately selected as the definitive input parameter configuration. Subsequently, employing these optimal input parameters, 50 runs were executed for each tree, and the distribution of primary branch counts was computed. The two critical parameters *Height*_*Segmentatioin* and *Cloud*_*Paramter* were set as default by recommendation for AdQSM.

## Results

### Tree instance and structure segmentation

#### Point cloud downsampling and skeletonization

While the tree instance segmentation algorithm fulfilled the need, it was not the focus of this study, and a more detailed evaluation will be available in a separate study.

**Fig. 3.**
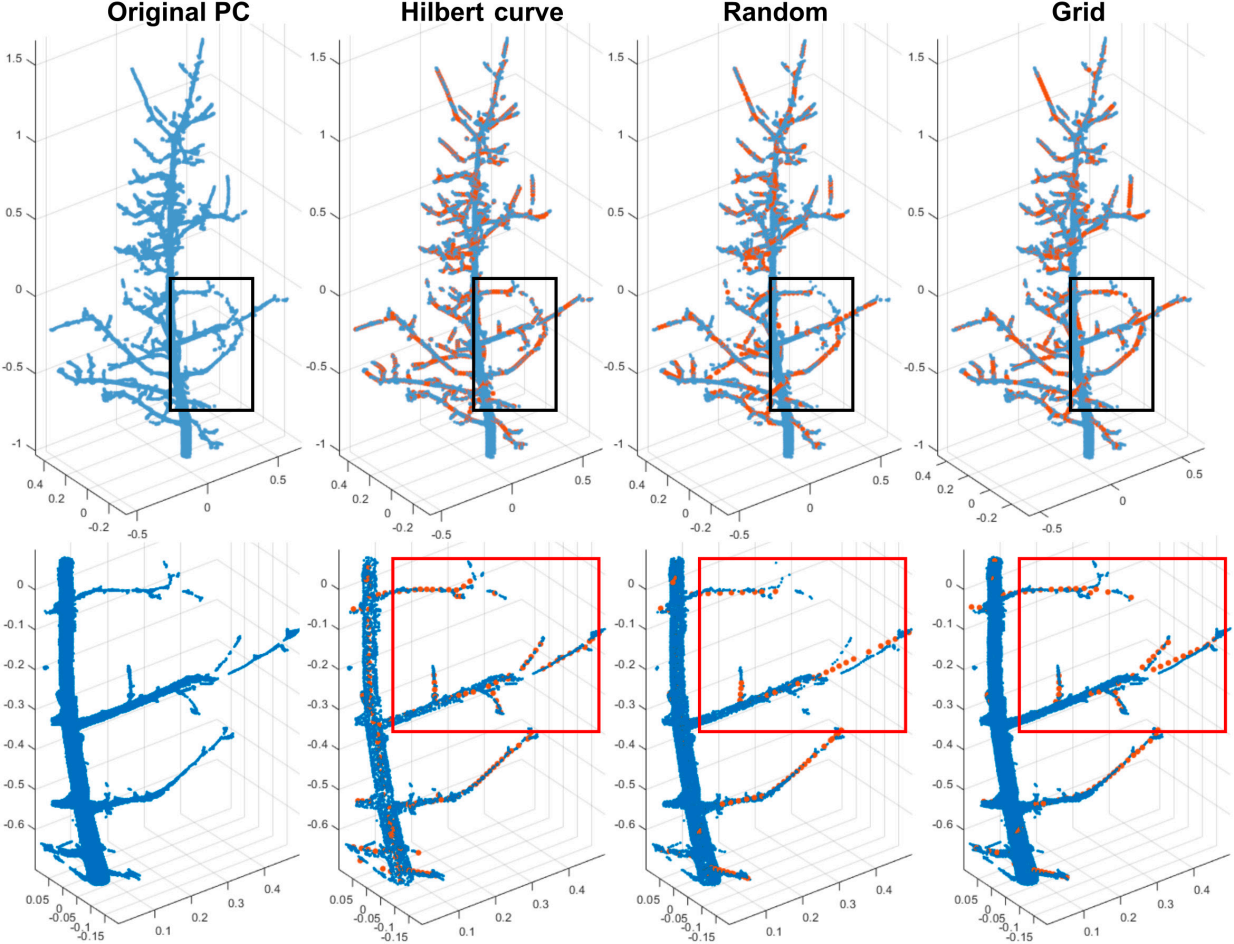
Downsampled point cloud and extracted skeletons using Hilbert curve, random, and grid downsampling algorithms.

Compared with traditional methods such as random and grid downsampling, the HC-based downsampling method was more effective and efficient in preserving the topological structure and geometrical features of original point clouds at the same downsampling rate (Fig. 3, top). In general, a total of 50,000 points per apple tree would allow tractable computation in the tree structure segmentation and architectural trait extraction. In this study, the average number of points for apple trees was 700,000, requiring an acute downsampling rate (i.e., 93%) that presented challenges to preserve needed information. The HC-based method aimed to balance the representativeness of points of different structure components with varying point densities during the downsampling process: retaining more points from a region with a lower point density (e.g., branches) and fewer points from a region with a higher point density (e.g., tree trunk itself) (see the histogram in Fig. S3). Therefore, the resultant point clouds could maximize the preservation of detailed topological information (especially at the branch level) and achieve the desired downsampling rate simultaneously. Neither random nor grid downsampling showed such an advantage. Random downsampling was nondeterministic, so downsampled point clouds might or might not contain all topological details for processing. Grid downsampling was limited by its inability to simultaneously maintain a high downsampling rate and preserve topological details. This limitation compromised the quality of results, particularly when high downsampling rates were employed. Specifically, the approach of grid downsampling that averages point distributions within each grid led to a misalignment between skeleton points generated from grid-downsampled point clouds and the original point clouds, a problem particularly pronounced when branches were sparsely populated with surface points.

Quantitative evaluation confirmed the superior performance of the HC-based downsampling method from qualitative observations (Table 3). This approach achieved on average 47,000 downsampled points per original apple tree point cloud, exhibiting the highest computational tractability among the three methods tested. Moreover, the HC-based method excelled in primary branch identification within the downsampled point clouds. It demonstrated a true-positive rate of 87%, surpassing both grid and random downsampling methods by 4% and 12%, respectively, while maintaining a false-positive rate of 5%, closely aligned with the lowest rate of 4% achieved by random downsampling. This balanced performance was attributed to the locality-preserving property of HC-based downsampling. In comparison, grid and random downsampling methods presented either high or low true- and false-positive rates concurrently due to their inability to optimize the representativeness of downsampled points.

**Table 3.** Branch recall information. The number of segmented branches is based on the skeleton extracted from the downsampled point cloud using Hilbert curve, random, and grid downsampling algorithms. The ground-truth branch count is 332.

Downsampling method	#Points			True positive—correctly segmented branches	False positive—wrongly segmented branches	Major of incorrect segmentation
	Min	Max	Mean			
Hilbert curve	45,428	51,306	47,554	288 (87%)	15 (5%)	Branch intersection and noise
Grid	45,489	52,579	48,560	275 (83%)	20 (6%)	Branch intersection and oversegmentation
Random	50,000	50,000	50,000	248 (75%)	13 (4%)	Branch intersection and oversegmentation

#### Skeleton connectivity refinement

**Fig. 4.**
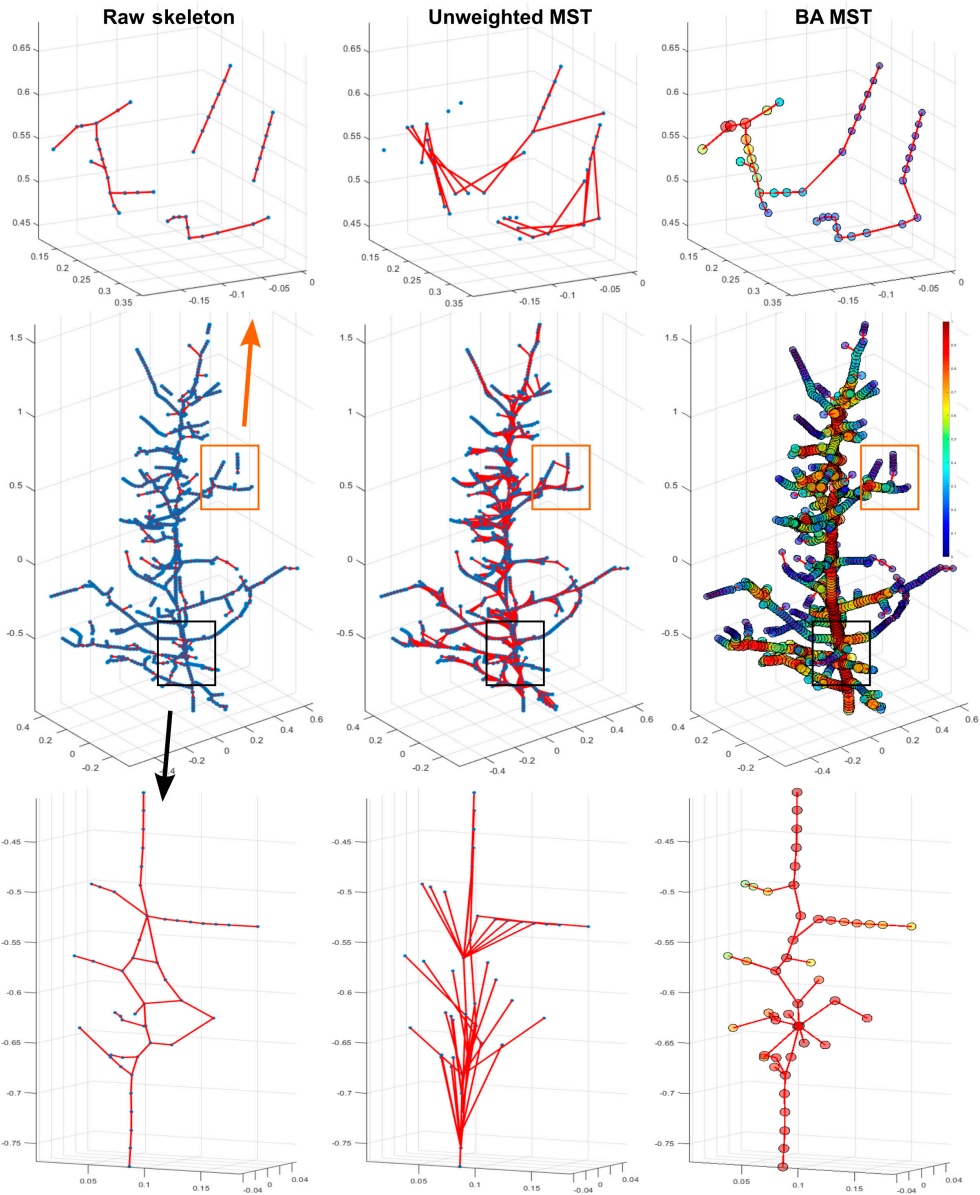
Skeleton connectivity and the optimal MST generated by unweighted graph and MST and BA graph and MST algorithm. Points and edges in unweighted MST have equal weight represented by the same blue dots. Points in BA MST have weights as an approximation of the local thickness estimated by naïve k-nearest neighbors (KNN) distance. Edges in BA MST have weights consisting of the weighted sum of the local thickness and Euclidean distance between two vertices.

By taking into account both the thickness and length cues, the developed BA graph could generate optimal MSTs where points were connected to obey the rules of the biological topology of a tree (Fig. 4). Raw skeletons largely maintained the topological structure of trees, but it was obvious that there were many defective connections such as disconnections between neighboring points and cyclic connections caused by errors in the FPS process. When they were used to form an unweighted graph for MST searching, these defective connections led to disruptions in tree skeleton topology and ultimately incorrect branch segmentation. This occurred because the MST searching method aimed to connect all vertices in a graph using the minimum number of edges (i.e., shortest tree). When edges were with the same weight, each graph vertex greedily established as many connections as possible without regard for topological correctness, leading to disordered connections (Fig. 4). These disordered connections originated from a point that could extend significantly to its surrounding points and destroy the topological structure. In contrast, the BA graph demonstrated the capability of producing the optimal MST with correct connections. The local thickness term guided the MST algorithm to find a path including more points with a larger thickness, which are likely to be trunk points. Simultaneously, the Euclidean distance term directs the MST algorithm toward finding the shortest path, which ensures that the connections are established sequentially between neighboring points and preserves the topological structure. By balancing the thickness and length information, the developed method utilized biological constraints to ensure the smoothness (therefore topological correctness) of continuous connections between skeleton points.

#### Trunk and branch segmentation

**Fig. 5.**
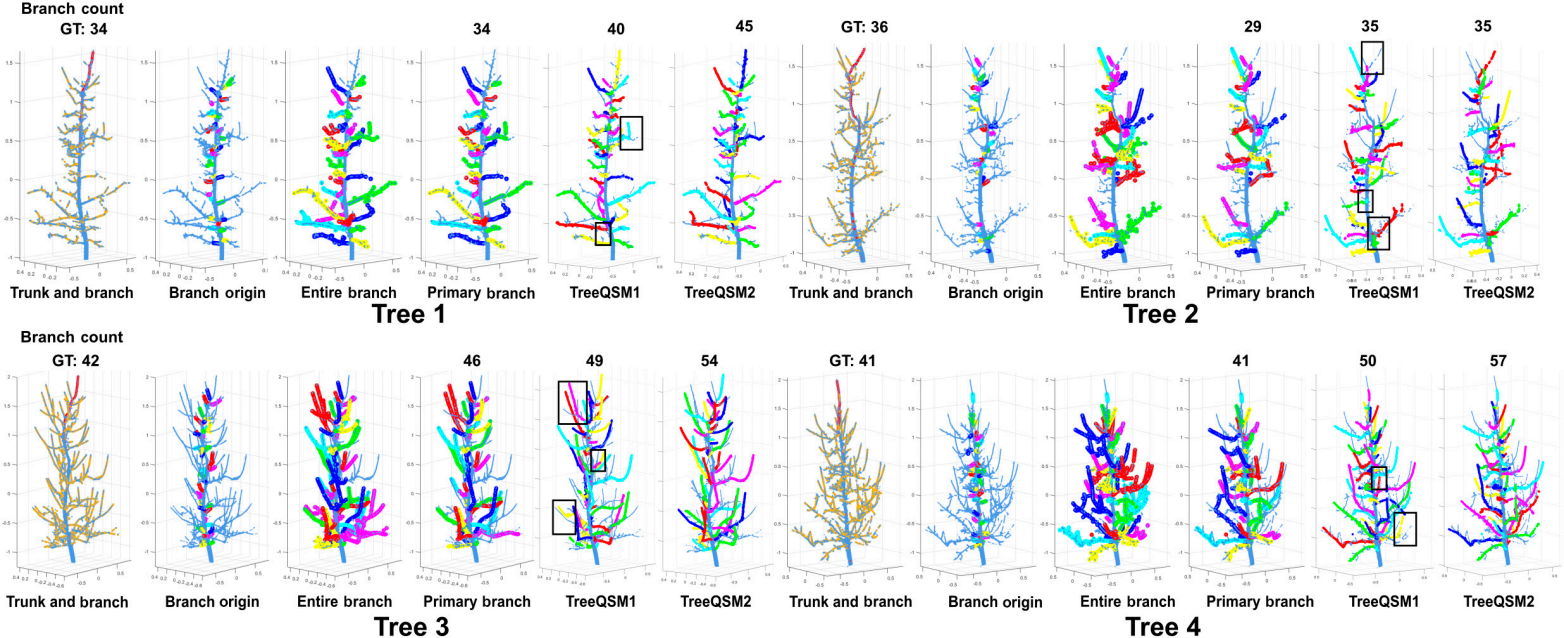
Trunk and branch structure segmentation results generated by the developed pipeline and TreeQSM. In particular, two results obtained from TreeQSM were selected out of 50 independent runs. Tree 1 and Tree 2 exhibit simpler branch structures with fewer branches, while Tree 3 and Tree 4 display more complex branch structures with a higher number of branches. The black boxes highlight the branch segmentation results that are inconsistent within TreeQSM runs.

The developed pipeline achieved a decent accuracy in trunk and branch segmentation (Fig. 5). Overall, the trunk segmentation was constantly successful and insensitive to downsampling strategies. This was because all three downsampling methods kept sufficient points of tree trunks for skeletonization and segmentation (see Fig. 3 for a zoom-in view and Fig. 5 for various trees). The branch segmentation was much more challenging than the trunk segmentation because branch skeletons typically contained less reliable points and deviated from the original point clouds. There were two potential reasons. First, the branch points were often incomplete because of the resolving power limitation of TLS, branch occlusions, and undesirable weather conditions. Second, the downsampling process further reduced the points for branches, especially those with high occlusions or small diameters. The sensing limitations could not be easily addressed by data processing, but the downsampling process played a critical role in branch segmentation (e.g., balanced true- and false-positive rates through HC-based downsampling). In point clouds downsampled by the HC-based method, most primary branch origin points were successfully grouped into different clusters. With the correct branch origin clusters, the BA graph with the MST algorithm was iteratively used for segmenting branches for the entire tree, resulting in an accuracy of 87% for branch segmentation. The primary branches were further segmented by finding the maximum path in individual branch MSTs.

The tube space for branch pruning was also important to the branch segmentation. An unsuitable tube radius might result in a disproportionate number of branch origin points, adversely affecting the number of branch origin clusters (Fig. S8). When the tube radius was excessively small, it led to the selection of minimal branch skeleton points, which the 3D DBSCAN algorithm was prone to categorize as noise. Conversely, an overly large tube radius resulted in an increased number of branch skeleton points used for clustering, thereby heightening the likelihood of erroneous cluster formation due to branch intersections and noise.

**Fig. 6.**
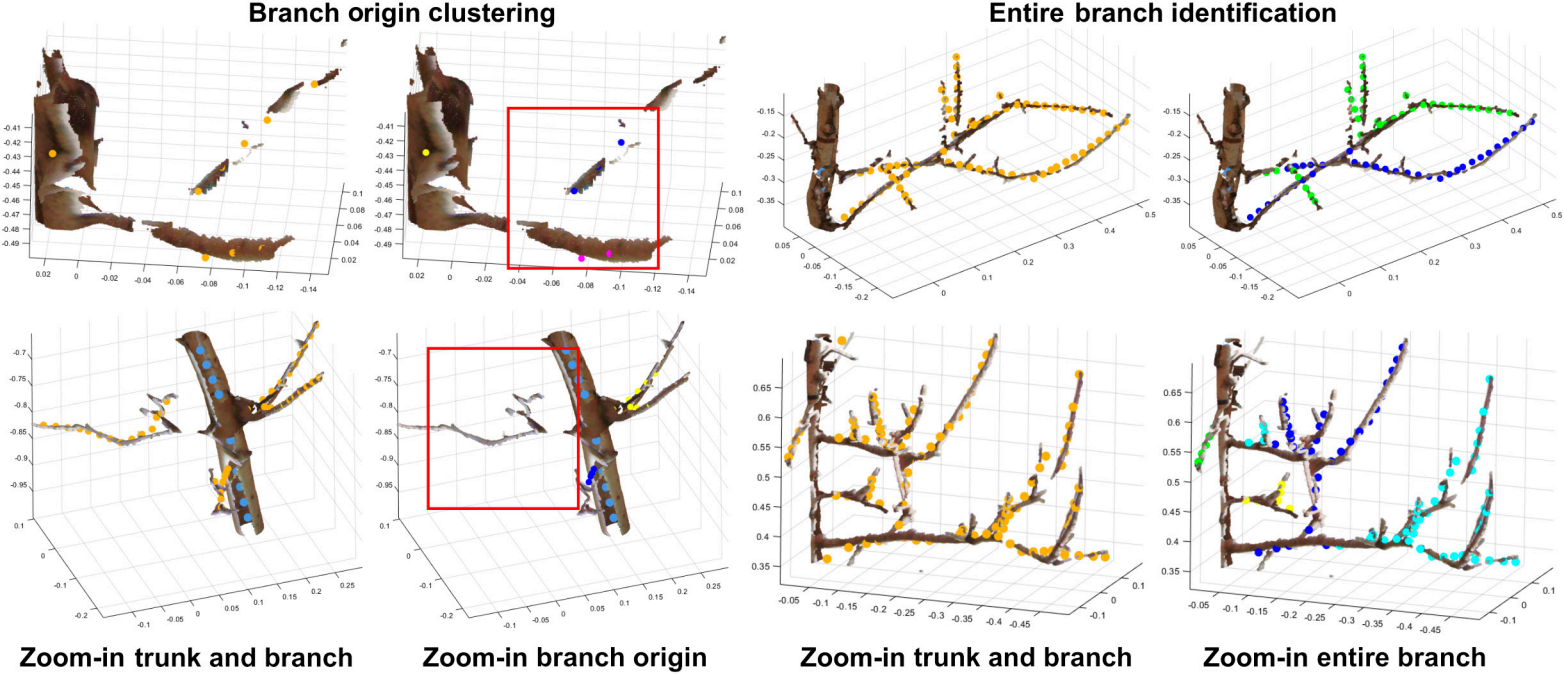
Representative of problematic branch clustering and segmentation. Under- and overclustering happened because gaps exist in the original point cloud. Incorrect entire branch segmentation happened because of branch intersection.

Two principal issues occurred with branch origin clustering due to absent points at the junction (see Branch origin clustering in Fig. 6). Overclustering arose when gaps manifested in the branch–branch junction, while underclustering was observed when these gaps were present in the trunk–branch junction. In addition, incorrect branch segmentation emerged with branches presenting complex patterns (see Entire branch identification in Fig. 6), typically observed at branch intersections where two or more branches entangled with each other. The MDM method, employed for post-processing, might not always correctly generate entire branches, especially with complex branch patterns. Consequently, this led to the failure of spectral clustering and ultimately unsuccessful corrections.

#### Trunk and branch refinement

**Fig. 7.**
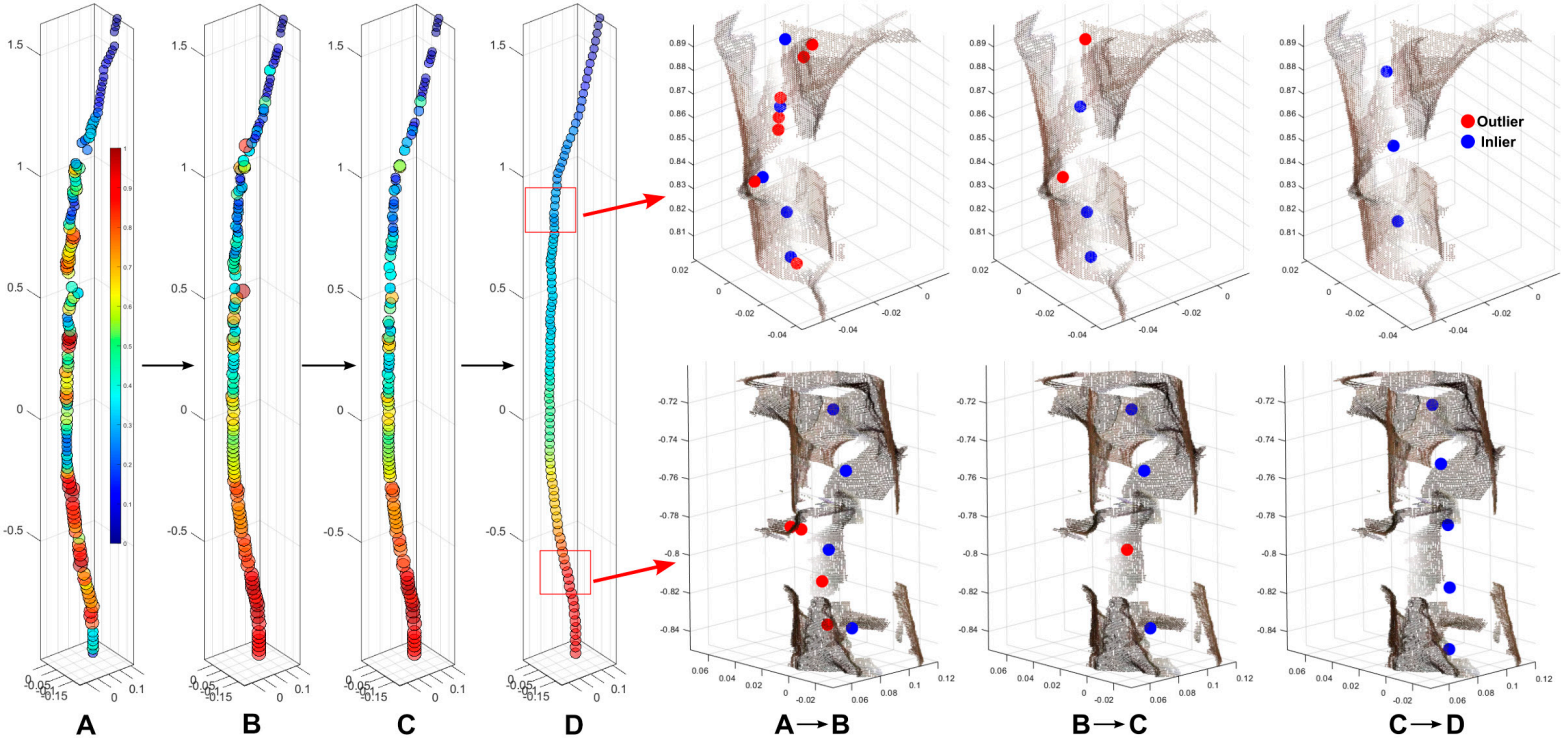
Trunk refinement illustration and results. (A) Coarsely segmented trunk skeleton with a weight estimated by the KNN distance. (B) Output from CPC optimization and local RANSAC. (C) Output from semi-global RANSAC. (D) Final output after cubic spline sampling.

The trunk and branch refinement process improved the overall quality of coarse trunk and branch skeletons in terms of topological correctness, centeredness, and spatial distribution (Fig. 7). The unrefined trunk and branch skeletons showed three critical issues (Fig. 7A). First, skeleton points were not well-centered and biased toward trunk and branch boundaries with most scanned points. Second, skeleton points were not uniformly distributed, presenting large gaps that could be problematic in successive analyses. Last, the thickness measured at the cross section of skeleton points (represented by color) showed unexpected fluctuations, violating the smoothness principle of trunk and branch diameter changing patterns along the length in biology.

The CPC-based optimization improved the thickness smoothness and centeredness of skeleton points by effectively optimizing the equal distance and variance objective function (Eq. 6). The local center correction operation further removed erroneous centers, resulting in a more topologically correct skeleton with a more consistent change of thickness (Fig. 7B). While the local center correction operation provided the context of trunk growing direction within a small segment (from Fig. 7A to Fig. 7B), there were still defective points because the CPC-based optimization was data-driven and fundamentally dependent on input point distribution. When noisy points took a large portion of the input, the results generated by the CPC-based optimization were biased toward those noisy points (Fig. 7B). The semi-global center correction operation successfully eliminated these deviated points in the context of overall trunk growth (from Fig. 7B to Fig. 7C). The processed skeletons showed an improved topological structure and thickness-changing pattern (Fig. 7C). Finally, the cubic spline sampling method ensured an evenly spaced distribution of points in the skeleton (Fig. 7D).

### Performance of architectural trait extraction

#### Quantitative evaluation

**Fig. 8.**
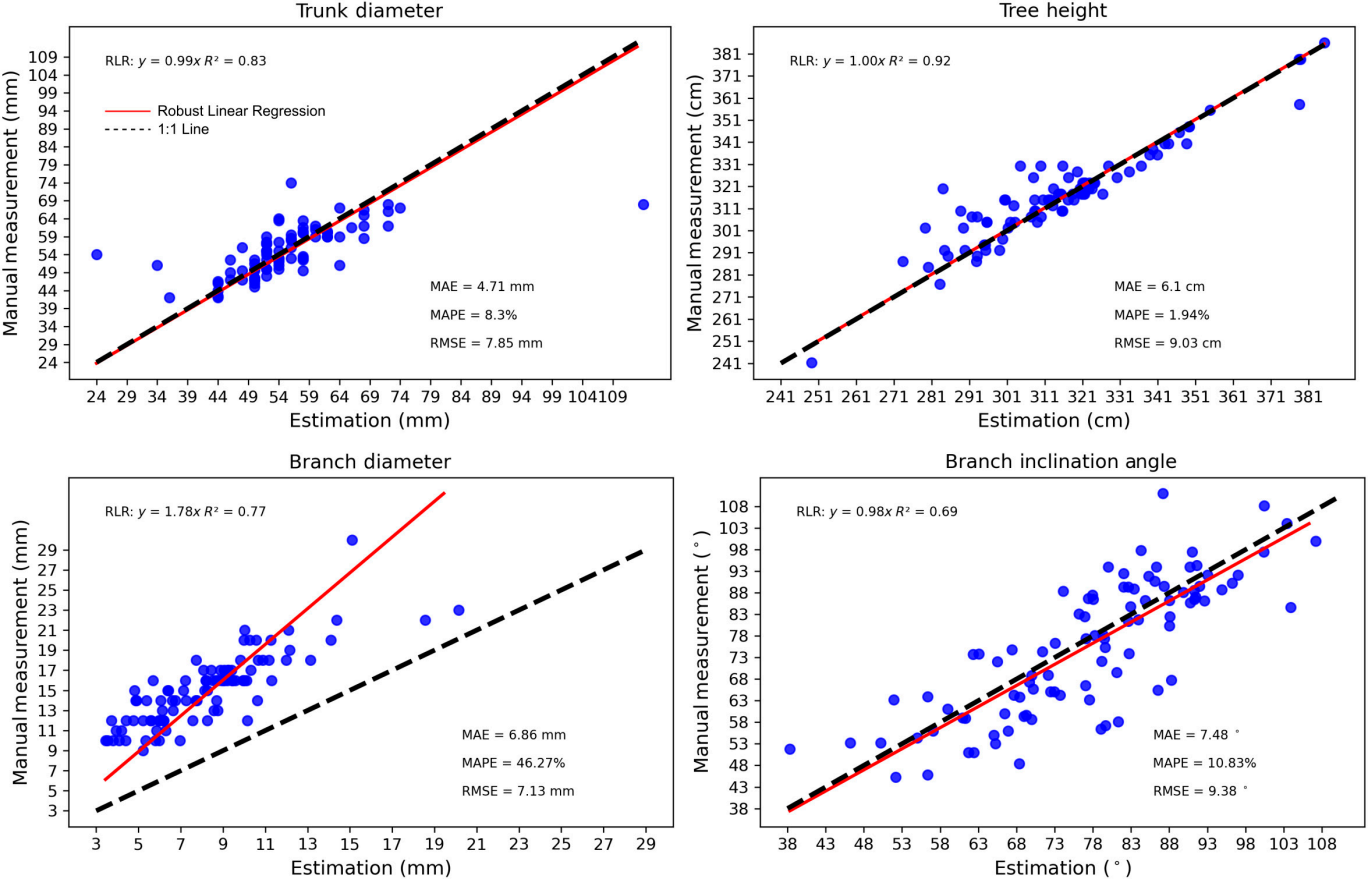
Trunk- and branch-level architectural trait estimation results and quantitative performance evaluation. RLR, robust linear regression.

The developed characterization pipeline was evaluated at the tree and branch levels (Fig. 8). Tree height and trunk diameter calculated using the developed pipeline were highly correlated (*R*^2^ = 0.92 and 0.83) with manual measurements with an MAE of 6.1 cm and 4.71 mm and a MAPE of 1.94% and 8.3%, respectively, indicating high accuracy of measuring architectural traits at the tree level. Few outliers were observed in trunk diameter measurement for various reasons such as the tree rootstock black cover interference and raw point cloud quality (Fig. S9). These presented challenges in developing a universal solution to address all potential edge cases. Additionally, the pipeline achieved a decent performance (*R*^2^ = 0.69, MAE of 7.48°, and MAPE of 10.83%) for measuring branch inclination angles with an overall correlation of 0.69, suggesting that the pipeline was able to provide good angle estimations with reasonable variations. The relatively lower correlation (i.e., 0.69) was primarily due to challenges in the field measurement protocol (Fig. S10). Consistent angle measurement in the field was too difficult because the exact 2D projection angle between branches and tree trunks could not be easily defined and accessed in practice. Consequently, the accuracy of manual measurements using a digital angle finder was compromised. Furthermore, branch inclination angles were computed in 3D space using point clouds, which differed from the field measurement protocol as a 2D projection. This measurement protocol difference could potentially increase the quantitative error. To investigate the effects of these challenges, the developed pipeline was also applied to point clouds of simulated trees. Experimental results showed a considerably higher correlation (*R*^2^ = 0.9) with reduced MAE and MAPE, suggesting that the relatively large measurement difference was due to imperfect manual measurements rather than the pipeline itself (Fig. S12). An additional experiment was conducted for branch inclination angle, using trees specifically planted for a dedicated breeding program (Figs. S1 and S11 and Table S1). For more comprehensive information and details regarding this experiment, please refer to the Supplementary Materials (Case Study: Characterization for Breeding Programs).

The pipeline exhibited large errors in branch diameter estimation primarily because of incomplete branch point clouds. The incompleteness of branch point clouds dramatically limited the accuracy of branch diameter measurement. This was because the diameter measurement relied on circle or ellipse fitting that required geometric completeness of points. If only a portion of points were provided, these fitting algorithms were likely to perform poorly and the estimated branch diameter tended to be smaller than the field measurements, resulting in a large diameter estimation error (e.g., large MAE and MAPE values). In fact, human operators could have difficulties of providing accurate diameter estimation using CloudCompare given highly incomplete point clouds (see point clouds in Figs. 6 and 7). However, the pipeline demonstrated a correlation (*R*^2^ = 0.77), indicating the efficacy of the CPC-based optimization to tackle incomplete point clouds for branch diameter measurement to a certain extent.

#### Comparison with TreeQSM and AdQSM

The distribution of primary branch counts has unveiled a noteworthy challenge—undesirable nondeterministic outcomes (Table 4 and Fig. S13). Even in the relatively straightforward task of counting primary branches, TreeQSM exhibited considerable variability in the number of primary branches (Fig. 5). This variability underscores the inherent stochastic nature of the model’s outcomes. Intriguingly, when applied to a simulated tree, TreeQSM demonstrated consistency in primary branch counting. This observation lends support to the notion that the processing performance bottleneck primarily stems from data quality rather than the underlying processing algorithms. Additionally, while AdQSM demonstrated consistent primary branch recall, it cannot address the reconstruction of the trunk–branch junction area (thereby the inaccurate branch diameter estimation) and is considerably sensitive to noise when reconstructing higher-order branches (Figs. S14 and S15 and Table S4).

**Table 4.** Primary branch recall information from the developed pipeline, TreeQSM, and AdQSM. The total manual measured number of primary branches is 332.

Method	Branch counts		
Min	Max	Mean
Developed	303	303	303
TreeQSM	383	405	393
AdQSM	377	377	377

In the agricultural sector, where activities such as fruit harvesting and branch pruning are heavily reliant on consistency and precision, the presence of nondeterminism poses a substantial challenge to the seamless integration of QSM methods like TreeQSM. Consequently, addressing the issue of nondeterminism becomes a pivotal endeavor, essential for unlocking their full potential in supporting large-scale agricultural applications. The unique characteristic of the developed pipeline lies in its ability to consistently segment primary branches and characterize the architectural traits of apple trees. This consistency and accurate reconstruction is of paramount importance as it fortifies the reliability and robustness of characterizations—an indispensable factor when considering the application of such methodologies within the apple industry, especially in the context of large-scale machinery operations.

## Discussion

### Overall pipeline parameterization and new opportunities to domain research

The developed pipeline demonstrated a satisfactory performance on 3D characterization of apple trees that exhibited variation in overall topological structure and branch patterns. This was made possible by leveraging improvements of all individual steps, including the HC-based downsampling strategy, the BA graph, and the CPC-based optimization process.

The HC-based downsampling strategy enabled the use of a limited number of points to represent a topologically complete downsampled tree structure, ultimately improving both the efficiency and accuracy of the characterization process. This downsampling method divided the entire point cloud of a tree into small grids and treated the number of points in each grid as the region density, allowing the curve to approximate trunk and branch regions based on point density. The “Iteration” parameter plays a critical role in the downsampling process, determining the resolution of the downsampled tree. A higher iteration level divides the original point cloud into smaller grids, resulting in a higher resolution downsampled tree that preserves features of sparse regions, such as occluded branch origin areas and tiny branches, more effectively.

The developed BA graph offered improved performance than conventional methods (e.g., unweighted graph) by incorporating biology constraints such as local thickness and Euclidean distance. The weights of these constraints were crucial in determination. A large weight for thickness guided the MST algorithm to select a path that included more points with a larger thickness, meaning that points in denser regions were more likely to be chosen, such as points in trunk regions and junction regions. However, it should be noted that the thickness of points was approximated by the region density, which might not always be an exact indicator of thickness due to data noise. Similarly, a large weight for length directed the MST algorithm to find the longest path, which could result in a long side branch being erroneously identified as part of the trunk.

While showing a certain efficacy of refining trunk and branch skeletons, the CPC-based optimization in this study was not as effective as the original research [60]. This was due to the significant difference in point cloud quality between the two studies. The original research evaluated the performance of the CPC-based optimization on a synthetic dataset with four separate perturbations, including different resolutions, noise, gaps (i.e., missing points), and varying point densities. However, the tree dataset used in this study contained a compounded perturbation. Therefore, the assumptions and parameters of the CPC-based optimization were examined more rigorously, and it was found that the CPC-based optimization was sensitive to model parameters and point geometry assumptions. In theory, the CPC-based optimization assumed taking a spherical point cloud as a segment to produce the best-optimized center because it optimized the minimum Euclidean distance and variance. However, in practice, trunk and branch cross-sections were cylindrical. To approximate the geometry assumption, the depth of trunk segments and branch cross-sections must be much smaller than their radii. Otherwise, the optimization was hard to converge.

The developed 3D characterization pipeline, due to its improved measurement accuracy, objectivity, and throughput, holds significant potential for employing TLS and geometry-based methods to assess fruit tree architectural traits at tree and branch levels. Specifically, for apple research and production, this pipeline would enhance crop load potential prediction, offering both accuracy and efficiency, crucial elements for precision crop load management. Estimating crop load potential during the off-season is key to guiding tree pruning and crop thinning during the growing season. Traditional mechanical pruning, relying heavily on human experience without quantitative data, often leads to imprecise pruning and consequential profit loss. The ability to accurately estimate apple crop potential enables precise mechanical pruning, optimizing which and how many branches to prune, thereby maximizing profits and ensuring high-quality apple production. Furthermore, knowledge of apple crop load potential aids in determining appropriate chemical spray applications in chemical crop thinning, achieving effective crop load reduction while minimizing environmental harm. Additionally, the pipeline was considered an effective tool for accurate and high-throughput measuring of branch inclination angles, potentially useful for large-scale genetics studies in tree architecture.

### Limitations of the developed characterization pipeline

#### Hardware limitations

Apple tree point clouds, captured using TLS under field conditions, furnish a detailed 3D depiction of the trees, facilitating the characterization of architectural traits at both tree and branch levels. Despite TLS’s ability to address occlusion issues inherent in 2D imaging systems, the acquired point clouds often lack comprehensive geometry, particularly for finer structures like branches. This is attributed to several factors, such as inadequate resolution and precision settings in the TLS configuration, harsh weather conditions like strong winds (causing issues in registering moving objects from multiple scans) and variable sunlight (causing issues in registering objects with color appearance variances from multiple scans), and the intricate intersections of tree branches. While enhancing the resolution and precision of TLS could improve accuracy, it prolongs scanning time, thereby constraining phenotyping throughput. One potential solution involves the use of a self-navigating robot platform equipped with mounted laser scanners to parallelize point cloud collection and registration, a method that would greatly benefit large field applications due to its efficiency [62].

#### Traditional algorithm limitations

The devised characterization pipeline demonstrated exceptional performance on simulated trees furnished with complete point clouds (Fig. S12), underscoring that the hurdle for granular branch trait characterization lies not within the geometry-based methods themselves but within the quality, particularly the completeness of the point clouds. The geometry-based analytical methods hinge on explicit data representation and geometric features in point clouds. Consequently, if the data representation is flawed or fails to provide sufficient geometric features, the geometry-based methods become inadequate to yield satisfactory results.

The point cloud registration, dependent on point geometry and color information, faces obstacles under field conditions, often struggling to generate a complete point cloud. Environmental factors such as wind and sunlight pose significant challenges to point cloud registration, inducing inconsistencies in the geometry and color of identical points. Wind-induced tree deformation results in inconsistent geometry information for the same points, instigating nonrigid transformations. Similarly, the Sun’s movement throughout the data collection process generates varying illumination and shadow effects, leading to changes in RGB color for the same area. These inconsistencies and nonrigidities complicate point cloud registration to seamlessly integrate points captured from multiple viewing angles. While the registration algorithm used in this study was designed to manage noise and outliers, points with inconsistent information were more prone to be deemed as noise and subsequently discarded. These hurdles complicate the attainment of a complete point cloud under field conditions using the existing registration algorithm. While the Laplacian-based skeleton method exhibits some resilience to missing points and variable point density, it becomes less reliable when the original point cloud contains substantial missing points and noise. In such circumstances, the extracted skeleton may not accurately reflect the tree’s true structure and topology. Close-proximity structures, already recognized as challenging for most point cloud processing algorithms [58], become even more problematic with complex branch intersections, exacerbating the limitations of the Laplacian-based skeletonization and leading to confusion at intersecting points and unreliable skeleton extraction. Furthermore, the quality of the extracted skeleton impacts branch segmentation, leading to under- and oversegmentation due to gaps appearing at trunk–branch or branch–branch junctions in the skeleton (see examples in Fig. 6). Missing points also present challenges in the CPC-based optimization for trunk and branch refinement. Although the RANSAC algorithm could potentially identify and exclude outliers in the refined trunk and branch skeletons, the quantitative estimation of trunk and branch diameter tends to be smaller than field measurements due to the incomplete surface of original point clouds. These limitations underscore the necessity of high raw data quality to yield top-tier results from geometry-based characterization methods.

Outliers observed in trunk diameter estimation were caused by three primary reasons (Fig. S9). First, the black cover removal operation in data preprocessing fundamentally eliminated a significant portion of the trunk at the bottom, introducing potential measurement errors. The trunk segmentation method started from the lowest point of a point cloud, which was always assumed to be the trunk point. However, due to the cropping, low branches could grow at a similar height as the cropped trunk, and their side branches could even grow toward a lower height, yielding a false starting point for the trunk segmentation method. Tree height estimation was slightly affected by this false starting point since the height difference between the false starting point and the true trunk point typically will not be large, whereas trunk diameter estimation could be dramatically affected. This was because the points for ellipse fitting were provided from a branch rather than the trunk and resulted in a considerably smaller estimation and thus a large error (Fig. S9A). Additionally, the trunk diameter measurement assumed that there was a constant distance buffer (i.e., 5 cm in this study) between the lowest trunk point and the lowest trunk–branch junction. When the distance was smaller than the constant buffer, the branch points would be clustered together with the trunk points, resulting in a much larger estimation (Fig. S9B). Second, the large number of missing trunk points essentially ruined the accuracy of the geometry-based trunk diameter estimation method. Last, although field measurements were used as reference measurements, practical challenges introduced possible error sources for the comparison between the pipeline-derived values and manual measurements for traits like branch inclination angle.

#### Learning-based methods in the future

The developed geometry-based pipeline’s characterization accuracy sets a precedent for the characterization of tree morphology at the tree and branch levels using data collected under field conditions. Further enhancement could be achieved using advanced learning-based methods. Learning-based point cloud registration methods have displayed several advantages over traditional methods, including robustness, generalizability, efficiency, and flexibility [63,64]. These techniques, through learning robust feature representations, manage to better handle noise, outliers, deformation, and data incompleteness. Moreover, such algorithms pave the way for integrating physical laws like tree stiffness models with data-driven methods to establish data-driven, mechanistic models.

Learning-based point cloud completion methods aim to infer missing parts that scanned data, due to sensor limitations and object occlusions, fail to reconstruct [65–68]. They are promising, given their ability to manage the high-dimensional and irregular nature of point clouds effectively. In contrast, geometry-based methods for point cloud completion, which rely on geometric properties like smoothness, curvature, and symmetry, typically use interpolation based on known data like neighboring points and surface normal. These approaches can be computationally expensive and may fail to capture complex geometries. Furthermore, these methods would require a sparse point cloud with uniformly spaced points, a condition seldom met in real-world applications.

Conversely, learning-based methods utilize deep neural networks to discern underlying patterns and structures in the data, allowing them to complete missing parts [65–68]. These networks are trained on large datasets, making them more robust to noise and able to generalize to varying scenarios and datasets. This adaptability is particularly valuable in real-world applications, where point clouds can differ in scale, complexity, and quality. Further, appearance and semantic information can be incorporated into neural networks, enhancing completion results and improving the accuracy and consistency of the completed point cloud, especially for objects with known shapes or structures, such as trees.

## Conclusion

In this study, a geometry-based data processing pipeline was developed to use high-resolution TLS point cloud for the characterization of trellised apple trees at both tree and branch levels. The developed pipeline accurately characterized tree-level architectural traits with consistent performance. Characterizing branch-level architectural traits proved more challenging because of incomplete point representation, although the pipeline maintained a satisfactory performance. The study also identified data representation quality as the primary obstacle limiting the performance of geometry-based analytical methods. Future research will concentrate on two key areas: (a) refining the current characterization pipeline to enhance its adaptability, generalizability, and efficiency, and (b) devising effective point cloud reconstruction and completion methods to provide new opportunities for improving characterization performance.

## Data Availability

The source code is available at the project GitHub repository (https://github.com/suptimq/Apple_Crop_Potential_Prediction/tree/master). Raw data used in this study will be shared upon reasonable request.

## Supplementary Materials

Supplementary 1 Case Study: Characterization for Breeding Programs Tables S1 to S4 Figs. S1 to S15
