# Supplementary material for: AppleQSM: Geometry-Based 3D Characterization of Apple Tree Architecture in Orchards
Source: Plant Phenomics. 2024 May 1;6:0179. doi: 10.34133/plantphenomics.0179 (PMC11077705; doi:10.34133/plantphenomics.0179)
Supplement: Supplementary 1 — Case Study: Characterization for Breeding Programs Tables S1 to S4 Figs. S1 to S15 [file plantphenomics.0179.f1.pdf]

AppleQSM: Geometry-based 3D Characterization of Apple Tree Architecture in Orchards
Supplementary Materials

Tian Qiu[1], Tao Wang[2], Tao Han[3], Kaspar Kuehn[4], Lailiang Cheng[4], Cheng Meng[5], Xiangtao Xu[3], Kenong Xu[6], and Jiang Yu[6*]

[1]School of Electrical and Computer Engineering, Cornell University, Ithaca, USA
[2]School of Mathematics, Tianjin University, Tianjin, China
[3]Department of Ecology and Evolutionary Biology, Cornell University, Ithaca, USA
[4]School of Integrative Plant Science, Cornell University, Ithaca, USA
[5]Center for Applied Statistics, Renmin University of China, Beijing, China
[6]School of Integrative Plant Science, Cornell University, Geneva, USA
[*]Corresponding author. Email: yujiang@cornell.edu

1 Case Study: Characterization for Breeding Programs

Eight three-year-old Gala-W apple trees grown on their own roots (derived tissue culture) in a Cornell Agritech Orchard (latitude: 42.880 N, longitude: 77.006 W) in Geneva, NY, USA, were used as an extra dataset to prove the versatility of the developed processing pipeline. These trees were planted at a spacing of 4.27 m (14 feet) by 0.91 m (36 inches) in 2020. Notably, these trees grow downward branches (Figure S1).

The developed apple tree characterization pipeline has also demonstrated effectiveness when applied to apple trees with altered shoot growth from upward to downward. Despite the unique and unconventional branch pattern, the pipeline has consistently yielded favorable results, showcasing a positive correlation between the traits under examination ($R^2 = 0.59$, MAE of 10.98°, and MAPE of 12.80%) (Figure S11). This achievement underscores the robustness and adaptability of the pipeline, promising a useful tool for high-throughput phenotyping of diverse tree growth habits that are relevant in apple genetic studies and breeding programs. It is worth noting that the error originated from the measurement protocol difference became more pronounced when trees exhibited an unconventional branch pattern.

Tables

Table S1: Summary of apple trees and point cloud data from the Cornell Agritech Orchard in Geneva.

Row ID	Cultivar	#Trees	Age	Height (cm)			Trunk Diameter (mm)			#Point Cloud Per Tree		
				Min	Max	Mean	Min	Max	Mean	Min	Max	Mean
Geneva	Gala-W	8	3	NA	NA	NA	NA	NA	NA	40730	138480	88922

Table S2: Summary of the meaning of symbols used in this study.

Symbol	Meaning
P	The input points
P_D	The downsampled points
$P^{'}$	The skeleton points after tree skeletonization
G_W	The biology-aware weighted graph
P_S	The optimal minimum spanning tree in G_W (i.e., the coarse skeleton points)
P_B	The branch origin clusters
P_S	The spectral clusters after the spectral clustering algorithm

Table S3: Critical pipeline parameters used in this study.

Section - Parameter Name	Meaning	(unit) Parameter Value
Downsampling - k	Hilbert curve order/iteration	7
Downsampling - N_S	#downsampled points	(#points) 50,000
Skeleton Extraction - ϵ	FPS radius	(m) 0.006 * $\lvert diagonalofpointcloudboundingbox \rvert$
Skeleton Refinement - d_{th}	Distance threshold	(m) 0.1
Skeleton Refinement - α	Balance weight	(m) 0.4
Trunk and Branch Segmentation - R	Tube space radius	(m) 6 * ϵ
Trunk and Branch Segmentation - eps	DBSCAN eps	(m) 1.5 * ϵ
Trunk and Branch Segmentation - N_{min}	DBSCAN minimum number for clusters	(#points) 3
Trunk and Branch Segmentation - d_{ext}	Projection distance threshold	(m) 0.06
Trunk and Branch Segmentation - $R^{'}$	Local region radius for CPC	(m) 0.02
Trunk and Branch Refinement - K_1	Semi-global center correction	(#points) 10
Trunk and Branch Refinement - M	#points sampled from spline	(#points) 100
Trunk and Branch Refinement - N	#points for radius estimate in spline	(#points) 3
Tree Level Architecture Traits - C_z	Height threshold	(m) 0.05
Branch Level Architecture Traits - K_2	#points for angle estimate	(#points) 4
Branch Level Architecture Traits - K_3	#points for radius estimate	(#points) 4

The most critical parameters to make the pipeline achieve the optimal performance are: 1) downsampling - k. This “Iteration” parameter determines the resolution of the downsampled tree. A higher iteration level divides the original point cloud into smaller grids, resulting in a higher-resolution downsampled tree, 2) skeleton extraction - ϵ which determines the number of skeleton points and the average distance between skeleton points. An increase in this value will lead to fewer skeleton

points, and 3) trunk and branch segmentation - R and DBSCAN parameters. A larger R will provide more points for DBSCAN clustering and may lead to an over-clustering issue.

Figures

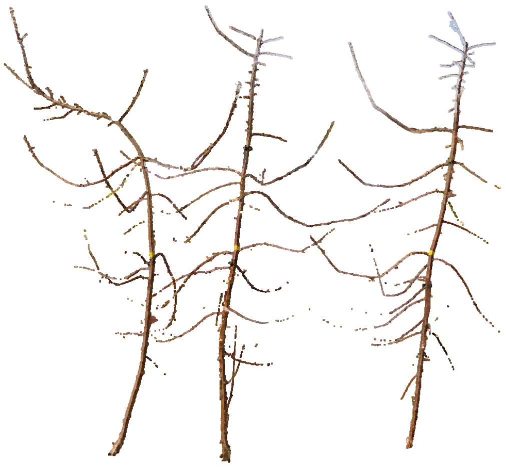

Figure S1: Representative trees from the Cornell Agritech Orchard in Geneva.

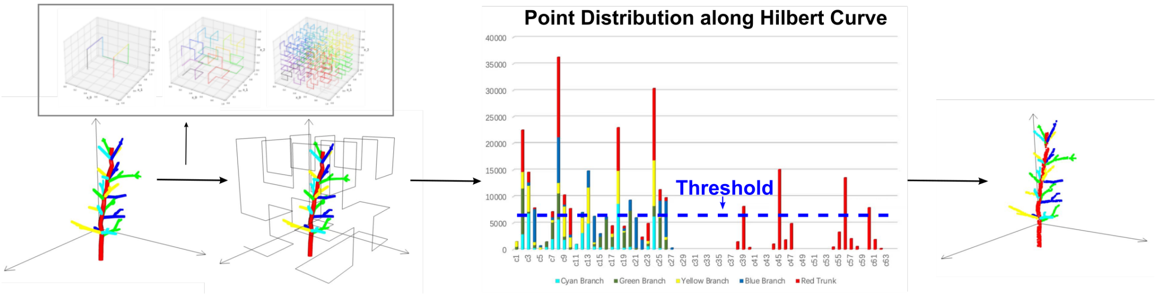

Figure S2: Illustration of Hilbert curve based downsampling method. Each individual branch was labeled by different colors to better demonstrate the process. The color coding was to intuitively illustrate the detail-preserving properties of the HC-based downsampling by visualizing how points belonging to different branches were sampled.

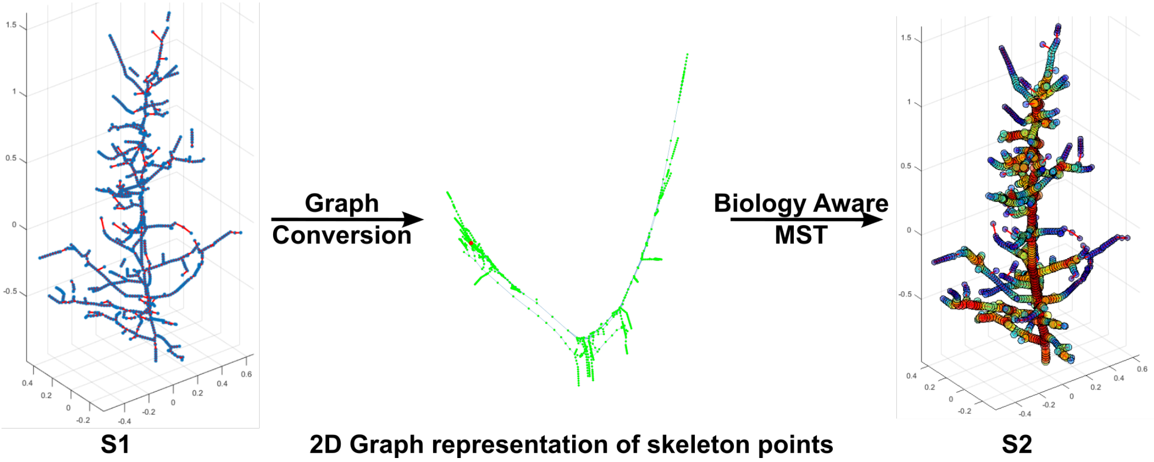

Figure S3: Biology aware graph and MST algorithm. S1 is the skeleton points with the raw connectivity (cycles and discontinuity exist in S1). A weighted graph was developed by taking each skeleton point as vertex and each connection as edges. The weight of edges is computed by a weighted sum of the total thickness of two vertices and Euclidean distance between two vertices. The MST algorithm was used to generate the optimal subgraph without cycles. S2 is the same skeleton points with the improved connectivity. The more red the vertex is the larger the weight is.

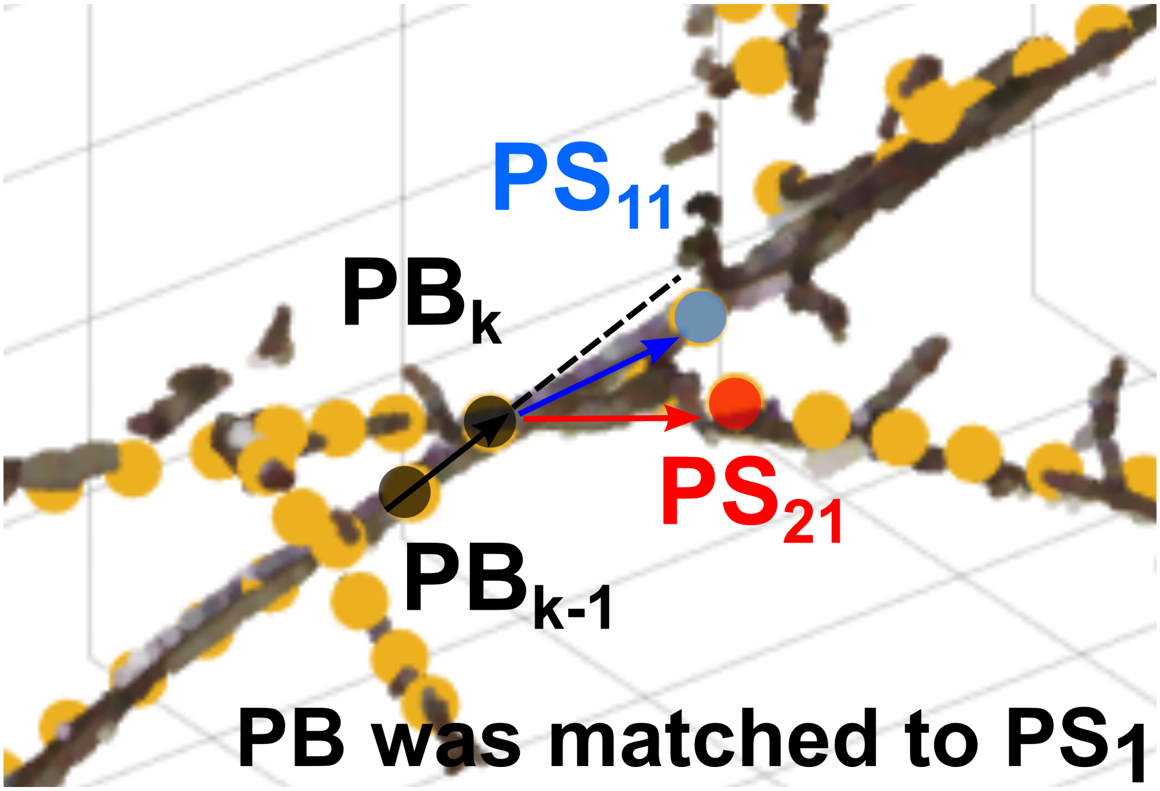

Figure S4: Illustration of the maximum direction matching (MDM) method for branch unique matching.

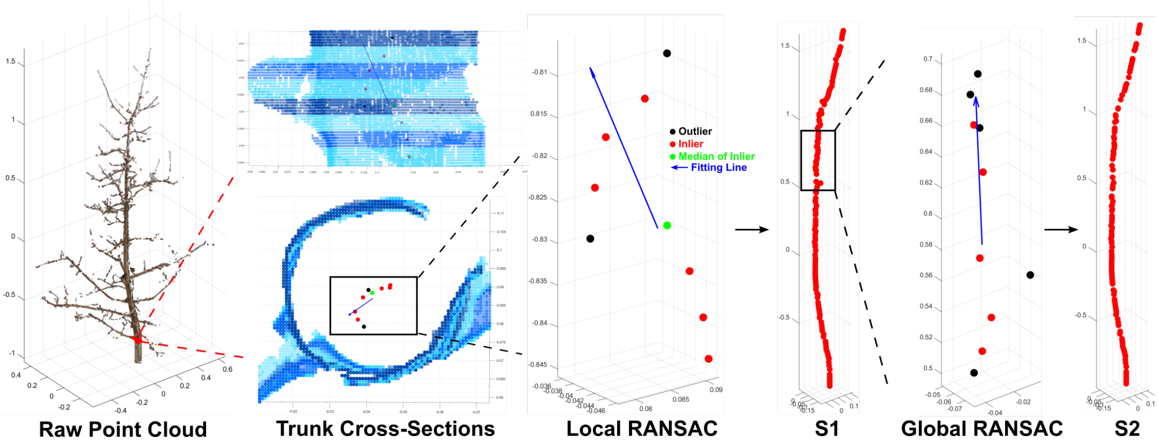

Figure S5: Illustration of trunk and branch refinement process. A cylinder pruning operation takes each coarse trunk skeleton point as center and points inside the cylinder were selected for the trunk refinement. The points were divided into cross-sections and CPC optimization was used to generate improved center points for each cross-section. Improved center points were post-processed by a local and global RANSAC to remove outliers produced because of irregular cross-sections. S1 and S2 are the improved trunk skeleton points output. Trunk skeleton points in S2 were used to fit a cubic spline and N points were uniformly sampled from the fitted spline as the final refined trunk skeleton points.

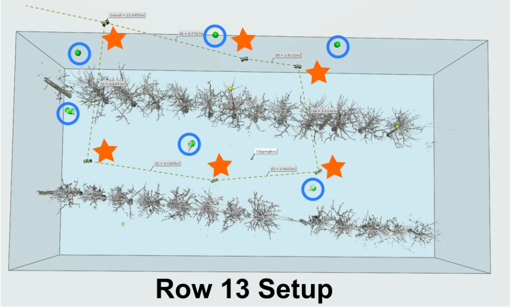

Scans Scan References

	Row 13	Row 15 and Row16
Maximum Point Error	2.9 mm	11.1 mm
Mean Point Error	2.5 mm	7.6 mm
Minimum Overlap	70.90%	48.10%

Scan Registration Statistics

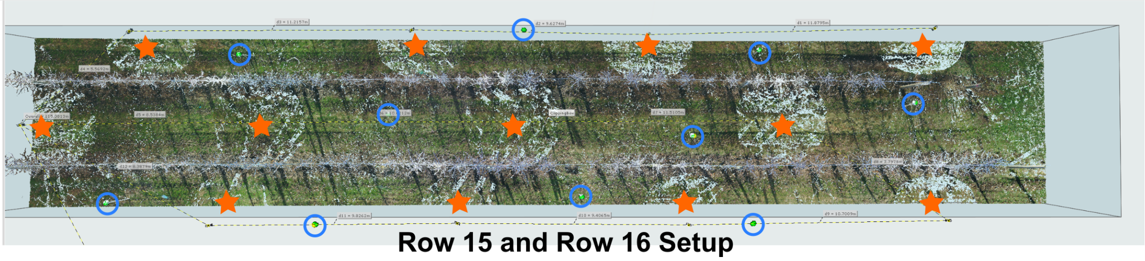

Figure S6: Illustration of scanning setup for row 13, row 15, and row 16. The distance between scans was shown on top of the dotted line (Zoom-in). The average distance of scans for row 13 is around 3 m, which presented a high overlapping between scans and led to low registration error. The scanning setup for row 15 and row 16 is a more standard setup to balance the scanning efficiency and data quality.

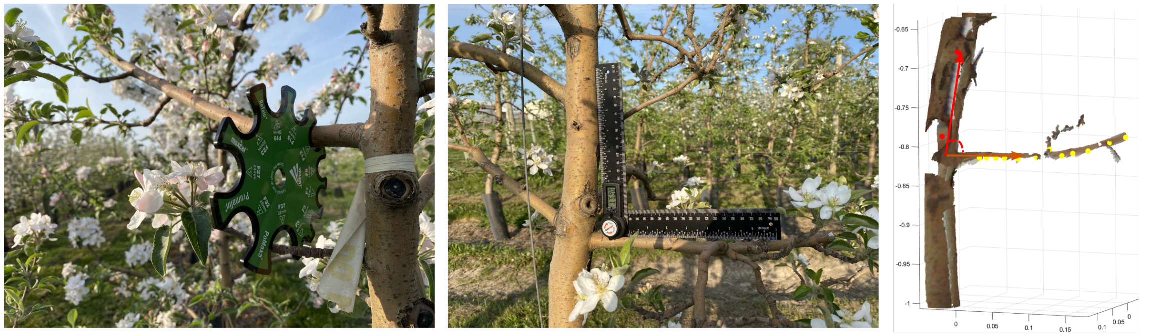

Figure S7: Illustration of branch level architecture trait field measurement and branch angle sensor measurement. In-field branch diameter was measured by an equilifruit disc (Valent LLC, USA) and in-field branch inclination angle was measured using a digital angle finder (CamRad 82305, Guilin GemRed Sensor Technology Co., Ltd, China) by placing it in the crotch. Sensor branch inclination angle was measured by computing the angle between two vectors in the 3D space.

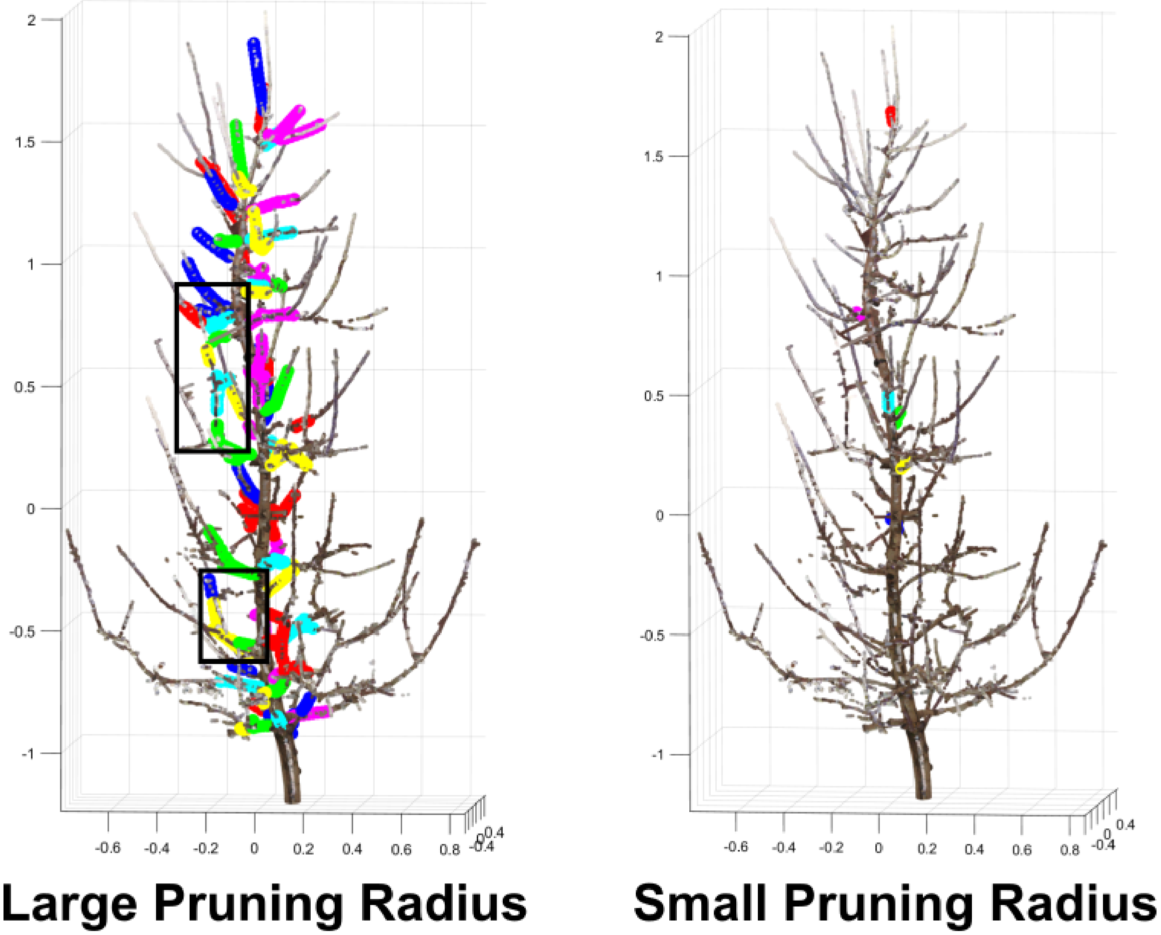

Figure S8: Branch root clustering results using improper sphere pruning radius. Using a large sphere pruning radius leads to over-segmentation issues, whereas using a small sphere pruning radius leads to under-segmentation issues.

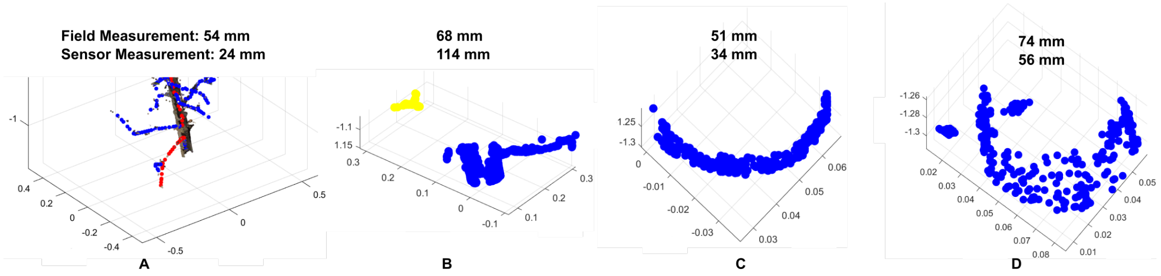

Figure S9: Showcase of the trunk estimation outliers. A: Red and blue points represent the trunk and branch skeleton points, respectively. The trunk skeleton starts from a wrong point because the side branch extends to a lower height than the trunk. B: Two clusters were produced by the 3D DBSCAN algorithm, and the blue cluster was the trunk cluster. However, a bunch of branch points were also included in the blue cluster, leading to a significantly larger trunk estimation. C: Only half of the trunk points exist in the original point cloud. D: The field measurement turns out to be inaccurate by using CloudCompare to manually measure the trunk diameter (i.e., 58.5 mm).

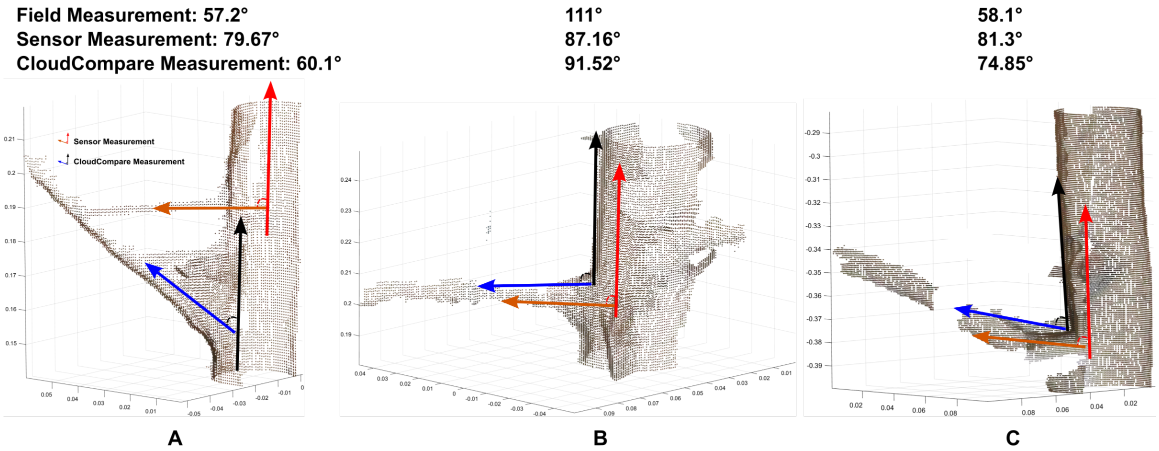

Figure S10: Showcase of failure and inconsistency in branch inclination angle estimation. A: The branch origin points were incorrectly identified because of noise so the estimation has a significant deviation. C and D: Different measurements in the inconsistent measurement approach.

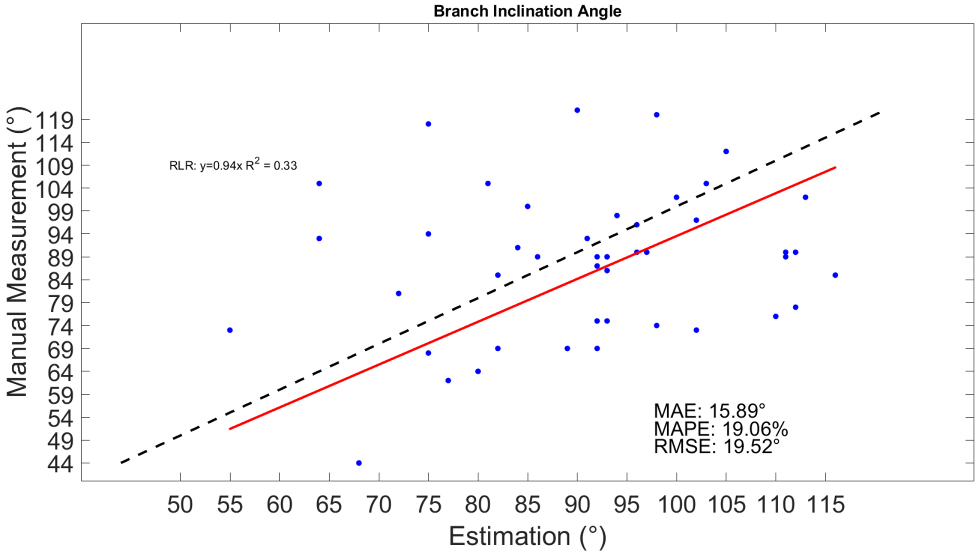

Figure S11: Branch angle estimation results of trees in the Cornell Agritech Orchard in Geneva.

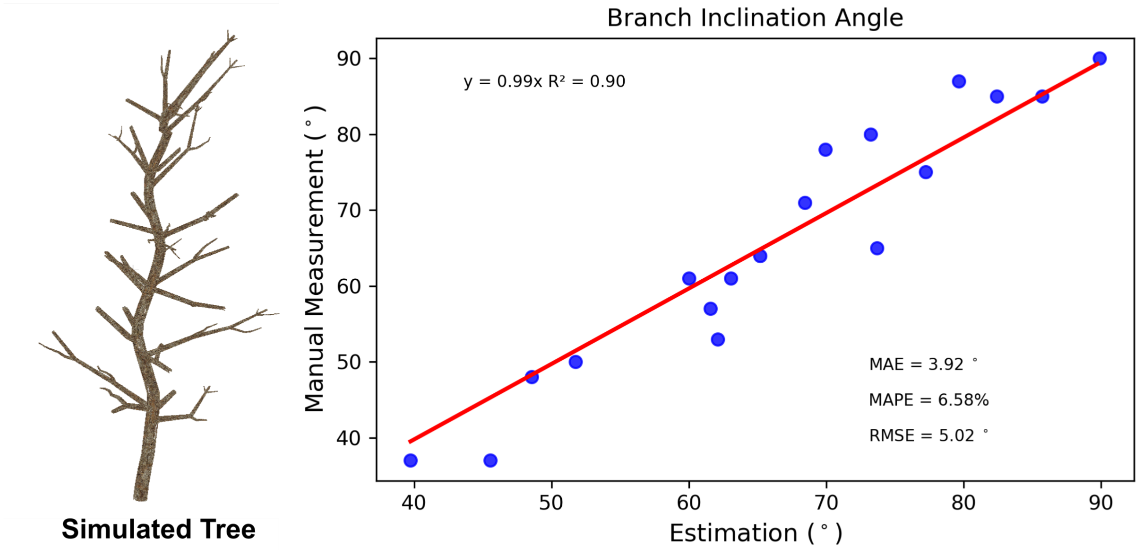

Figure S12: Branch angle estimation results using an artificial tree.

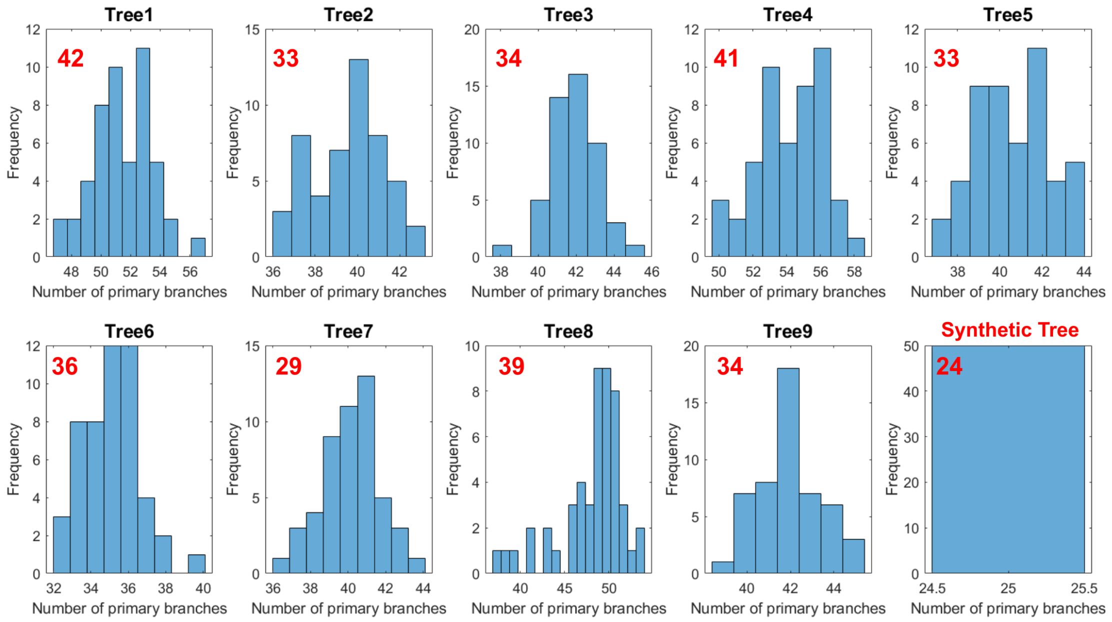

Figure S13: The distribution of the number of primary branches characterized by TreeQSM in 50 independent runs. The ground truth was indicated by the red number

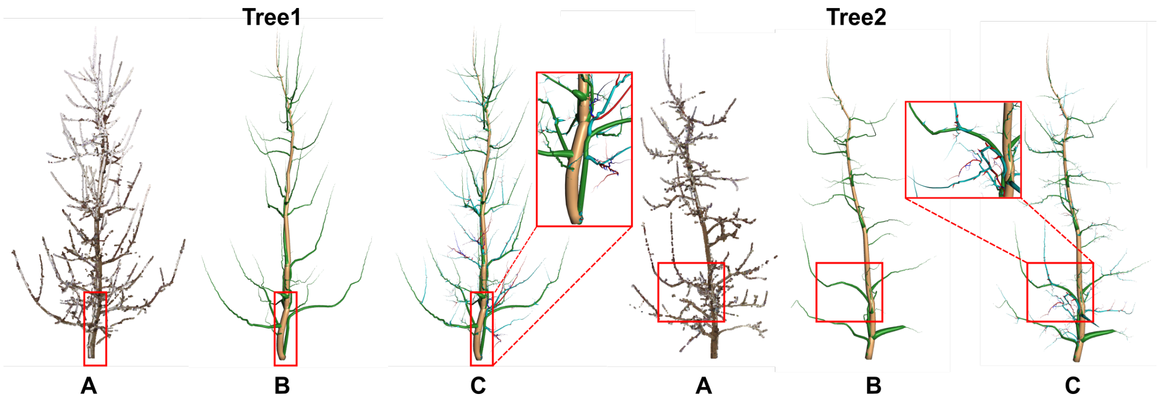

Figure S14: Representative of AdQSM reconstruction results. A: The original tree point cloud. B: The simplified AdQSM reconstruction result with primary branches only. C: The full AdQSM reconstruction result with all order of branches. Green: 1st-order branch, Cyan: 2nd-order branch, Red: 3rd-order branch, and Blue: 4th-order branch.

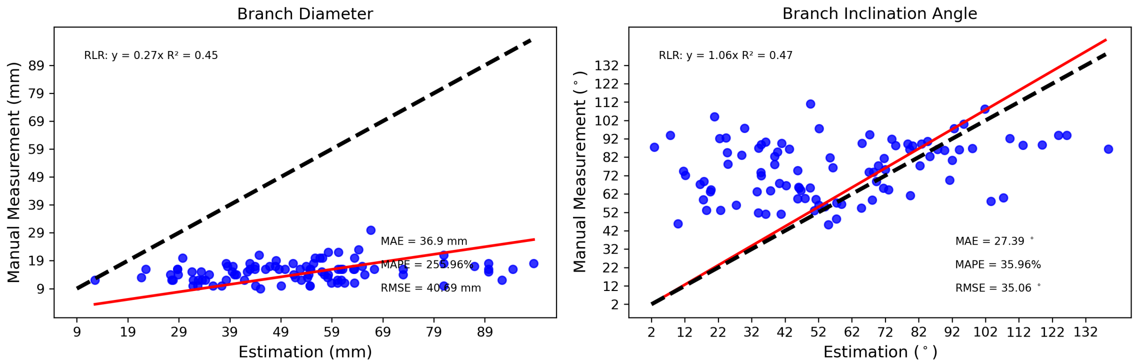

Figure S15: Branch diameters and inclination angles extracted using the AdQSM method.

Table S4: The number of segmented branches for eachh order from AdQSM.

Tree ID	#1st-order branches	#2nd-order branches	#3rd-order branches	#4th-order branches	#5th-order branches
Tree1	42	341	334	92	22
Tree2	44	330	382	110	0
Tree3	44	295	327	132	0
Tree4	48	318	408	198	0
Tree5	48	310	324	0	0
Tree6	38	292	328	143	0
Tree7	41	237	191	0	0
Tree8	37	267	270	0	0
Tree9	35	203	297	168	0
